# Analyzing Clonal Variation of Monoclonal Antibody-Producing CHO Cell Lines Using an *In Silico* Metabolomic Platform

**DOI:** 10.1371/journal.pone.0090832

**Published:** 2014-03-14

**Authors:** Atefeh Ghorbaniaghdam, Jingkui Chen, Olivier Henry, Mario Jolicoeur

**Affiliations:** 1 Canada Research Chair in Applied Metabolic Engineering, École Polytechnique de Montréal, Montréal, Québec, Canada; 2 Department of Chemical Engineering, École Polytechnique de Montréal, Montréal, Québec, Canada; Technical University of Denmark, Denmark

## Abstract

Monoclonal antibody producing Chinese hamster ovary (CHO) cells have been shown to undergo metabolic changes when engineered to produce high titers of recombinant proteins. In this work, we have studied the distinct metabolism of CHO cell clones harboring an efficient inducible expression system, based on the cumate gene switch, and displaying different expression levels, high and low productivities, compared to that of the parental cells from which they were derived. A kinetic model for CHO cell metabolism was further developed to include metabolic regulation. Model calibration was performed using intracellular and extracellular metabolite profiles obtained from shake flask batch cultures. Model simulations of intracellular fluxes and ratios known as biomarkers revealed significant changes correlated with clonal variation but not to the recombinant protein expression level. Metabolic flux distribution mostly differs in the reactions involving pyruvate metabolism, with an increased net flux of pyruvate into the tricarboxylic acid (TCA) cycle in the high-producer clone, either being induced or non-induced with cumate. More specifically, CHO cell metabolism in this clone was characterized by an efficient utilization of glucose and a high pyruvate dehydrogenase flux. Moreover, the high-producer clone shows a high rate of anaplerosis from pyruvate to oxaloacetate, through pyruvate carboxylase and from glutamate to α-ketoglutarate, through glutamate dehydrogenase, and a reduced rate of cataplerosis from malate to pyruvate, through malic enzyme. Indeed, the increase of flux through pyruvate carboxylase was not driven by an increased anabolic demand. It is in fact linked to an increase of the TCA cycle global flux, which allows better regulation of higher redox and more efficient metabolic states. To the best of our knowledge, this is the first time a dynamic *in silico* platform is proposed to analyze and compare the metabolomic behavior of different CHO clones.

## Introduction

Monoclonal antibodies (mAbs) are among the largest segment of today's therapeutic proteins market, with a 21% annual increase rate in launching into clinical trial [1]. Indeed, although CHO cells is now the major cell line used industrially with culture and production protocols that have been largely optimized [2], mAbs production at high quantities and of high quality, e.g. with defined glycosylation profile, still has to be achieved. Among many factors affecting mAbs quality, the stability with time of high producing level CHO cell clones with enhanced endogenous pathways (e.g glutamine synthetase (GS) gene) [3], and presenting a prolonged cell viability level due to the over-expression of some cytoplasmic proteins (e.g. chaperones such as Hsp70 and Hsp27) is highly critical [4]. Moreover, media composition and culture conditions, as well as their management along with culture duration, have to be optimized to achieve not only the objective of desired cell productivity and viability but also mAbs quality specifications [5]. Ultimately and within this context, efficient process control strategies, fed through on-line and off-line analyses, may allow seeking and maintaining desired optimal conditions with time. However, due to the large number of variables and decision steps associated with the development and the identification of a stable high-producer cell line, it is a highly challenging and time consuming process [6], [7]. Indeed, high-throughput screening approaches are normally used for clone selection, but there is a risk of performance discrepancy during scaled-up and manufacturing [8]. Therefore, only a knowledge-based strategy capable to detect at each step desired and undesired cell traits, as well as to extrapolate its behavior at the process scale, can efficiently guide and accelerate cell line screening works. Indeed, such level of knowledge has thus to be based on an adequate description of cell behavior in a managed environment. In that context, various “omic” approaches have been applied to cell line characterization. Clonal variations in rat fibroblasts [9] and hepatoma cells [10] were first reported and revealed differences in growth characteristics under both oxygen deficient and aerobic culture conditions. Proteomic and genomic studies on various NS0 [11], [12], [13] and murine cell lines [14], and of their recombinant derivative clones, allowed to clearly demonstrate that clones differing in their mAb productivities also differ in the abundance of proteins involved in cellular functions such as energetic metabolism, mAb folding/assembly, and cytoskeletal organization. The issue of clonal variation in recombinant CHO cells has also been largely addressed. Early works compared clones for their growth and morphological aspects, and showed altered cell morphology and different sub-population spatial organization types between clones when grown on agar [15], [16]. Clone-specific variations at the functional genetic level were also extensively described. It has been reported that high- and low-producer CHO-mAb subclones differ mainly in their DNA fragment sizes where high numbers of differentially expressed genes were identified [17]. Analyses at the proteomic level also revealed that different clones show different behaviors at different culture phases such as at mid-exponential and stationary [18]–[22]. The effect of culture conditions on different CHO cell clones, with respect to cell growth and productivity, was also investigated at reduced temperature [23]. Regarding specific productivity, different enhancing effects of low culture temperature were observed in different clones. Recently, a metabolomic study focusing on clonal variations in response to culture condition variation has been conducted [24]. Comparing clone-to-clone changes, beside specific productivity, strong variations in cell density, nutrient uptake and metabolic generation patterns were also detected. Indeed, various fluxomic approaches [25] have been developed to estimate metabolic fluxes rates, such as using labeling techniques [26]–[30] and metabolic mathematical models [31]–[36]. Using isotope labeling experiments, metabolic flux analysis (MFA) techniques and mathematical models, different metabolic patterns in CHO cell clones were observed such as a higher metabolic efficiency as a result of lower by-products production. Taken together, these works have significantly improved our knowledge on CHO cell behavior, as well as our conviction on the need for developing tools allowing a more in-depth capacity to describe cell metabolic behavior. In that context, kinetic models, when they describe transient behaviors, can serve as *in silico* platform enabling either intuitive or counter-intuitive metabolic flux exploration. In this work, we have further developed a kinetic-metabolic model for CHO cells. The model, which is based on cell energetic and redox states [36], was implemented with metabolic regulation aspects and then applied as an *in silico* platform to the characterization of clonal variation comparing a parental CHO cell line to its high- and low-producer derived clones. High- and low-producer clones, engineered with the inducible cumate gene-switch expression system [37], [38] were cultured in shake flask cultures, under both induced and non-induced conditions. The model was calibrated on experimental data of extra- and intracellular metabolites. In the present work, we thus present a descriptive model as well as evaluating its predictive capacity.

## Materials and Methods

### Ethics statement

All cell culture protocols were approved by the ethics committee of the Ecole Polytechnique.

### CHO clones and culture

CHO clones that stably produce a recombinant monoclonal human anti-CD20 at different specific productivities (high- and low-producer) were provided by Viropro International Inc. (Montreal, Quebec, Canada). These cells were derived from CHO-Cum2 cells and stably express the reverse cumate transactivator, as described in details by Mullick et al. 2006 [37]. Cells were seeded at 2×10^5^ cells mL^−1^ in 300 mL of a protein-free medium in 1-L shake flasks, and cultured on a shaker (150 rpm) in a humidified incubator at 37°C and 5% CO_2_. The medium used was a customized chemically-defined SFM4CHO medium (Hyclone, Utah, USA) supplemented with 4 mM glutamine (Hyclone, Utah, USA, cat. # SH30034), 30 mM glucose (Sigma, Oakville, Canada, cat. # G8270), and 0.05 mg mL^−1^ dextran sulphate (MW: 500000, Sigma, Oakville, Canada, cat. # D7037). For the comparative study, the parental clone, together with high- and low-producer clones, were cultured in duplicate. High- and low-producer clones were cultured both in the presence and in absence of cumate, the latter serving as non-induced control. In case of induction, 1 μg mL^−1^ of cumate was added after 48 hours of incubation, to trigger the recombinant protein expression. It should be mentioned that no visible effects on morphology or growth rates were reported for mammalian cells cultured at a cumate concentration below 200 μg mL^−1^ [37]. Cell culture samples were taken every 24 h for cell counts, biochemical assays, and quantification of amino acids and human IgG, the recombinant mAb. Samples were centrifuged at 300 *g* for 5 min to remove cells, and supernatant samples were stored at −20°C for further analysis. Cultures were monitored for a total of 6 days.

### Analytical methods

Cell density was determined by cell counting using a hemocytometer, and cell viability was estimated using the trypan blue (sigma, Oakville, Canada cat. # T8154) exclusion method. The concentration of glucose, lactate, glutamine and glutamate in the culture supernatant were determined using a dual-channel immobilized oxidase enzyme biochemistry analyzer (2700 SELECT, YSI Inc. Life Sciences, Yellow Springs, OH, USA), using calibration buffers provided by the manufacturer. Ammonia concentration in supernatants was assayed by an enzymatic kit with respect to manufacturer technical instructions: Ammonia Assay Kit (Sigma, Oakville, Canada cat. # AA0100). NAD(P) and NAD(P)H were also extracted and assayed by an enzymatic kit with respect to manufacturer technical instructions: NAD(P)/NAD(P)H Quantitation Kit (BioVision, CA, USA, cat. # K337-100). mAbs concentration was quantified using an enzyme-linked immunosorbent assay (ELISA). First, 96-well plates (Costar) (Fisher Scientific, Burlington, Canada, cat. # 3795) were coated with a goat anti-human IgG1 (H+L) solution (Jackson Immuno Research, PA, USA, cat. # 109-165-003) diluted to 2.5 μg mL^−1^ in 50 mM sodium carbonate (Fischer Scientific, Burlington, Canada, cat. # S263-1), and incubated at 4°C overnight. Then, the blocking of non-specific sites was carried out by adding PBS solution containing 1% casein. After incubation for 1 h at 37°C, either samples or standards diluted in PBS-casein were added in triplicate to each well and incubated for 1 h at 37°C. After the plates were incubated 1 h at 37°C, peroxidise-conjugated affinipure fragment Goat anti-human IgG (Jackson Immuno Research, PA, USA, cat. # 109-035-003) (1∶10,000 dilution) was added to each well, and the plates were incubated for 1 h at 37°C. After each of the previous steps, the wells were washed three times (PBS with 1% w/v Tween 20). Finally, the reaction was revealed by 3,3,5,5′-Tetramethylbenzidine (TMB) (Sigma, Oakville, Canada, cat. # T0440) and stopped after 15–20 min by adding 1 N hydrochloric acid, and the plates were red by an automatic plate reader at 450 nm using a Victor^3^ V microplate reader (Perkin-Elmer, Vaudreuil-Dorion, Canada). The analysis of amino acid concentrations was performed on an Agilent 1290 UPLC system (Agilent technologies, Montreal, Quebec, Canada) coupled to an Agilent 6460 triple quadruple mass spectrometer (Agilent technologies, Montreal, Quebec, Canada), following methods previously described [39], [40]. The underivatized amino acids were separated by a 2.1×150 mm ZIC™-Hilic column (3.5 μm, 200 A, PEEK) (Merck SeQuant, Peterborough, Canada) and 2.1×20 mm ZIC™-Hilic guard column (5 μm, 200 A, PEEK) (Merck SeQuant, Peterborough, Canada) at a column temperature of 35°C and injection volume of 5 μL. The mobile phase buffer contained 20 mM HCOONH4 (Sigma, cat. # 74314) at pH 4. The mobile phase A was 10% of the mobile phase buffer in water, and the mobile phase B was 10% of the mobile phase buffer in acetonitrile (ACN) (Sigma, cat. # A3396). The mobile phase B was linearly decreased from 90% to 35% in 19 min, then was increased to 90% in one minute and held at 90% for 15 min at a flow rate of 0.1 mL min−1. The Agilent 6460 triple quadruple mass spectrometer (Agilent technologies, Quebec, Canada), equipped with a Jet stream electrospray ion source (Agilent technologies, Quebec, Canada), was used for the analysis of amino acids in negative ion mode. The other parameters: Gas temperature of 350°C, Gas flow rate of 9 L min−1, Nebulizer pressure of 45 PSI, sheath gas temperature of 350°C, sheath gas flow rate of 10 L min−1, capillary votage of 3 kv. An internal standard solution which contains 2 μM Homoarginine (Fisher cat.# AC169090010), 2 μM homophenylalanine (Sigma cat.# 294357) and 2 μM Methionine-d3 (CDN isotope D1292) was used as internal standard for quantification. The MRM transition and retention time of each amino acid is listed in Table S1. It should be noted that commercial standards of every nutrients and metabolites were also used to establish calibration curves along with each series of analysis. Finally, extraction efficiency and compounds stability were determined using internal standards.

### Respirometry test

Respirometry assays were performed as described by Lamboursain et al. [41]. Briefly, 3 mL of cell suspension containing at least 5×10^6^ cells were inoculated in a 10-mL borosilicate glass syringe (Sigma, Oakville, Canada), in which the plunger was substituted by an Ingold pO_2_ probe (Mettler Toledo, Montreal, Quebec, Canada). At low cell densities, a volume of cell suspension containing 5×10^6^ cells was collected and centrifuged, and the pellet was re-suspended in a total of 3 mL of spent media. The respirometer was kept at 37°C and magnetically agitated (60 RPM) to ensure the homogeneity of cell suspension. Dissolved oxygen was recorded by an acquisition system (Centris, Longueuil, Quebec, Canada).

### Extraction of intracellular metabolites

For intracellular metabolomic analysis, 5×10^6^ cells were obtained daily, washed twice with cold PBS and extracted with 400 μL of 80% cold methanol in the presence of 0.2 g of sand (Sigma, Oakville, Canada, cat. # 274739). After 10 min on dry ice, the mixture was vortexed and then sonicated in ice and water for 5 min. Suspensions were then centrifuged at 4°C for 7 min at 21,000 *g*. The supernatants were then transferred to a clean tube as extracts. Pellets were re*-*extracted as mentioned above with 200 μL of 50% cold methanol and 200 μL of cold water. At each extraction, supernatants were combined with the first extract and stored in −80°C prior to analysis.

### Energetic nucleotide concentrations

Extracts were filtered through 0.2 μm filters (Millipore, Etobicoke, Canada) before analysis. Nucleotides in CHO cells extracts were analyzed using a 1290 UPLC system coupled to a 6460 triple quadruple mass spectrometer (both from Agilent Technologies, Montreal, Quebec, Canada). Nucleotides were separated by a Symmetry C18 column (150×2.1 mm, 3.5 μm) (Waters, Milford, USA) equipped with a Security C18 guard-column (Waters, Milford, USA 10×2.1 mm, 3.5 μm) by the ion-pair method, as previously described [42]. DMHA (N,N-dimethylhexylanine, Sigma, Oakville, Canada, cat. # 308102) was used as an ion-pair reagent to improve the signal-to-noise ratio with positive ionization mode. The mobile phase consisted of Buffer A: 10 mM ammonium acetate, 15 mM DMHA at pH 7.0, and Buffer B: 50/50% (v/v) acetonitrile, 20 mM NH_4_OAc at pH 7.0. Mobile phase flow rate was set at 0.3 mL min^−1^ with the following gradient: 0–10 min at 10% B, 10–20 min at linear gradient from 10 to 30% B, 20–21 min at linear gradient from 30 to 60% B, 21–26 min at 60% B, 26–27 min at linear gradient from 60 to 10% B and 27–35 min at 10% B. External standard curve was used for quantification. The Agilent 6460 triple quadruple mass spectrometer (Agilent technologies, Quebec, Canada), equipped with a Jet stream source (Agilent technologies, Quebec, Canada), was used for the analysis of nucleotides in positive ion mode. The mass spectrometer parameter were 100 ms scan time; 350°C gas temperature; 7 L min^−1^ gas flow rate; 30 PSI nebulizer pressure; 350°C sheath gas temperature; 12 L min^−1^ heath gas flow rate and 3500 V capillary voltage. The data was recorded in MRM mode with the mass spectrometer conditions listed in Table S2.

### Organic acid and sugar phosphate concentrations

Extracts were filtered through 0.2 μm filters (Millipore, Etobicoke, Canada) before UPLC–MS/MS (Agilent, Montreal, Quebec, Canada) analysis equipped with a Hypercarb column (100×2.1 mm, 5 μm) and a Hypercarb pre-column (2.1×10 mm, 5 μm) (Thermo Fisher, Burlington, Canada), as previously described [43], [44]. Mobile phase consisted in Buffer A: 20 mM ammonium acetate at pH 7.5, and Buffer B: 10% (v/v) methanol in water. Flow rate was set at 0.3 mL min^−1^ using the following gradient: 0–5 min at 10% A, 5–10 min at linear gradient from 10% to 20% A, 10–20 min at linear gradient from 20% to 100% A, 20–30 min at 100% A, 30–32 min at linear gradient from 100% to 10% A and 32–40 min at 10% A. The Agilent 6460 triple quadruple mass spectrometer (Agilent technologies, Quebec, Canada), equipped with a Jet stream source (Agilent technologies, Quebec, Canada), was used for the analysis of sugar phosphates and low molecular organic acids in negative ion mode. The mass spectrometer parameter were 100 ms scan time; 300°C gas temperature; 7 L min^−1^ gas flow rate; 35 PSI nebulizer pressure; 400°C sheath gas temperature; 12 L min^−1^ heath gas flow rate and 3500 V capillary voltage. Data were recorded in MRM mode with the mass spectrometer conditions listed in Table S3. The external standard curve was used for quantification.

### Model development

**Figure 1:**
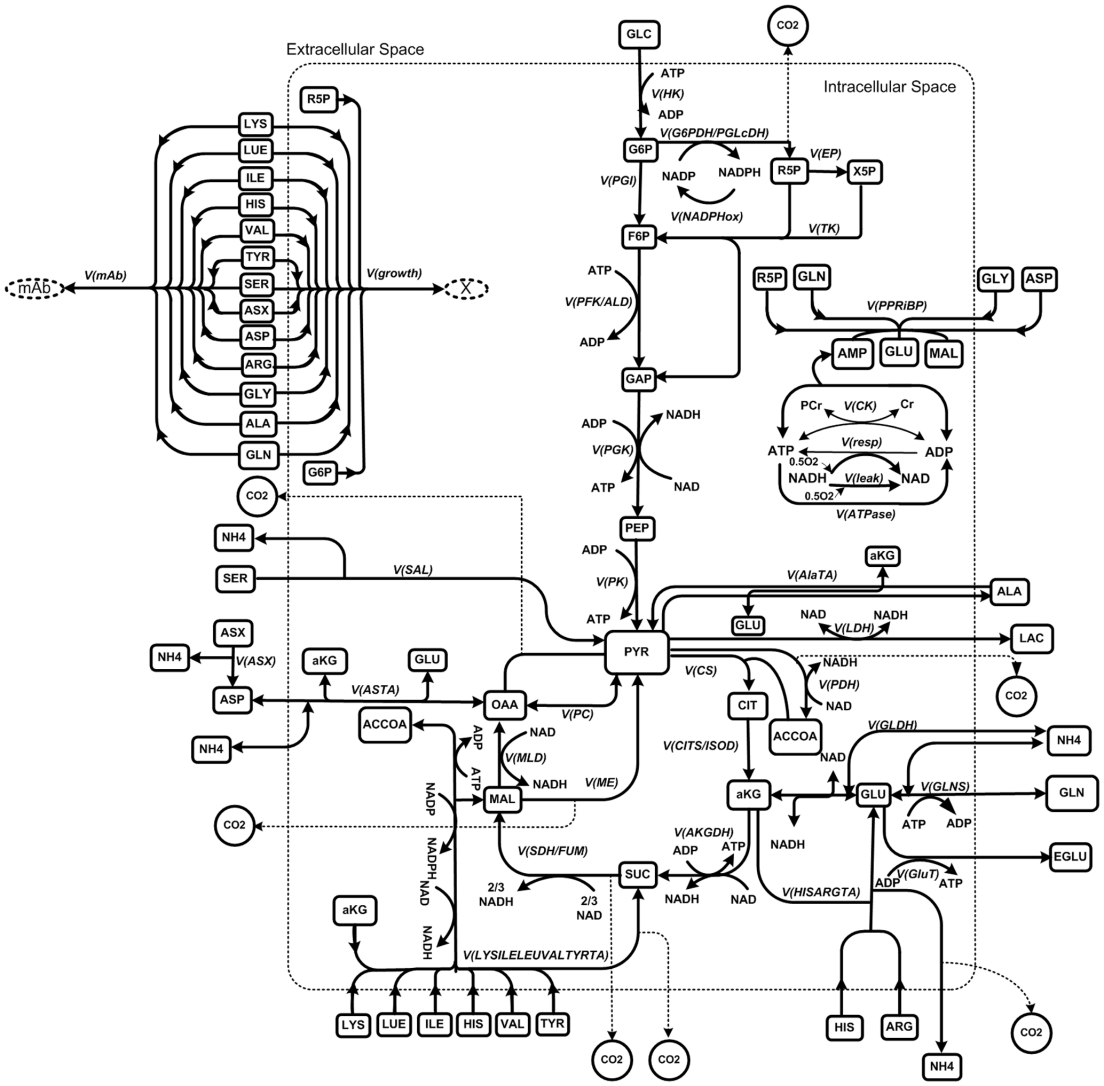
The metabolic network considered in the model.

The global structure of the mathematical model developed and presented here is based on a previous model describing CHO cells central metabolism [36]. Details concerning the model (transient mass balances, parameters, etc.) are provided in the Supporting Information; with the description of the biochemical reactions considered in the model metabolic network (Table S4), fluxes' kinetic formulation (Table S5), state variables and initial conditions (Table S6), affinity constants (Table S7) and maximal fluxes' rates (Table S8). In the present work, the descriptive precision as well as the predictive capacity of the model were improved by including catabolic pathways of amino acids metabolism along with other biochemical pathways (glycolysis, pentose phosphate pathway, TCA cycle, glutaminolysis as well as cell respiration) providing carbon skeletons to the central metabolism (Figure 1). For simplification purposes, amino acids are pooled into 3 groups channeled through TCA intermediates such as succinate, oxaloacetate and α-ketoglutarate. The other entry points for amino acid carbon skeletons are lumped to pyruvate. A special care was taken to preserve all stoichiometric relationships while lumping and/or combining reactions. In addition, we also further described the cell specific growth rate from its precursor's building blocks by considering G6P (leading to phospholipids and organic phosphate compounds), R5P (to DNA, RNA and nucleotides), and extracellular glutamine together with other amino acids (to proteins) (reaction 34, Tables S4 and S5). Cell growth is described from the main cell building blocks for which experimental data were available; thus excluding precursors of lipids. This approach, although reductive, allowed describing cell growth with culture time from the major anabolic pathways that are logically expected to affect growth behavior, as previously demonstrated [36]. Furthermore, a description of the cell-specific recombinant protein production rate from the mAb composition in amino acids is incorporated into the model. Extracellular amino acid concentrations are included individually in the kinetic expression for both the cell specific growth rate and mAb productivity. For simplification purposes and because of a lack of available data in literature as well as experimentally, a single affinity constant value is used for each amino acid, either as a substrate for biomass formation or antibody production, except for glutamine. Indeed, experimental data show that cell growth stopped specifically upon depletion of glutamine, while it has not limited antibody production. Consumption rate of each precursor for the synthesis of biomass or recombinant protein is calculated as proposed by Martens [45], considering the stoichiometry of precursor metabolites (Table S4, reactions 34–35). The mass balance on amino acids thus includes their production (where it applies) and their consumption for anabolic needs for growth and production as well as their contribution toward energy production through TCA cycle.

The stoichiometric coefficients of the respective biosynthetic equations were taken from literature [27]. The global metabolic network is presented in Figure 1, and a detailed summary of each flux reactions is given in Table S4. Only amino acids measured in this work are considered in the model. For amino acids, only extracellular pools were considered except for glutamate; extracellular glutamine is directly converted to intracellular glutamate, and intracellular glutamate exchange for extracellular glutamine is also considered to account for the management of nitrogenous sources as the culture enters the plateau phase. Intracellular glutamate is channeled through TCA cycle via a bidirectional exchange for α-ketoglutarate, as reported by Nolan et al. (2001) [34]. From experimental data obtained in this work, extracellular aspartate concentration showed low constant values as the culture reaches the plateau phase, which suggests a possible exchange of intracellular oxaloacetate for extracellular aspartate, a phenomenon that has thus been described in the model. Finally, it is assumed that at low extracellular glutamine level, the cells take up extracellular alanine; an activation term based on a threshold concentration for extracellular glutamine was thus included in the model.

### Description of flux kinetic regulation

**Figure 2:**
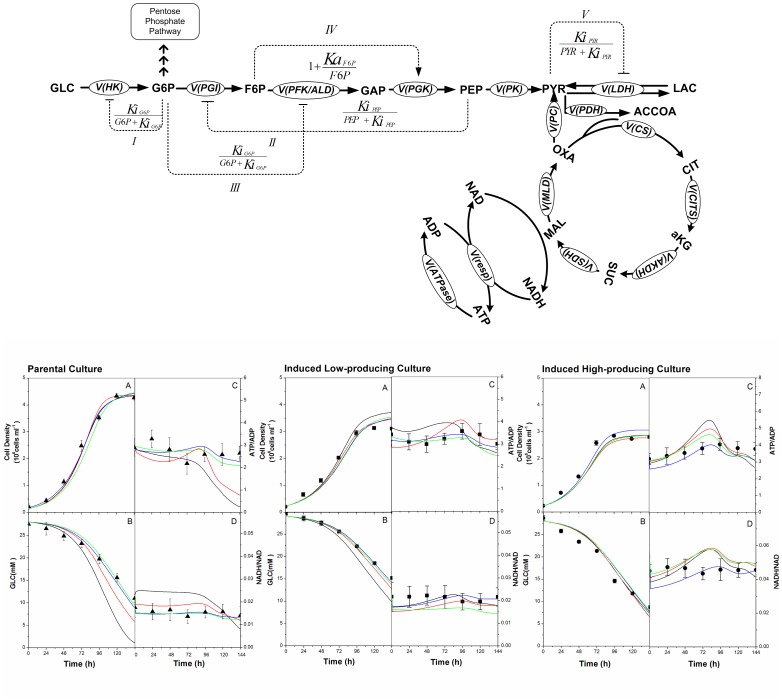
Regulation scheme of the model with enzymes activation or inhibition. Symbol “↓” indicates activation and “⊥” inhibition. Glycolytic enzymes are either inhibited 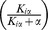
or activated 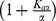 by an effector “α”. The corresponding activation/inhibition terms are labeled as I, II, III, IV, and V. The bottom diagram represents model simulations for parental, induced low- and induced high-producer cell lines with no regulation (solid black line), with the addition of term I (solid red line), with the addition of terms I and II (solid blue line), and the addition of all terms (solid green line). Experimental data are represented by triangles (parental culture), squares (induced low-producing culture), and circles (induced high-producing culture) for cell density (A), glucose (B), ATP-to-ADP ratio (C), and NADH-to-NAD ratio (D). Error bars are standard deviations from duplicate flasks. Error bars are standard deviations for duplicate cultures.

Mathematical formulations of metabolic flux kinetics have been determined based on a previous work [36] or adapted from Segel (1993) [46], both for their biological mechanistic representation and for the model capacity to simulate experimental data for another CHO cell line in bioreactor cultures. Michaelis-Menten type kinetic formulation was applied considering substrates, co-factors [47], [48], energetic nucleotides ratios, as well as inhibitors and activators when required as described in literature (*brenda-enzymes.info* [48]). ATP-to-ADP ratio has been reported to be maintained, in metabolically healthy cells, at a ratio around 10∶1 [49], NADH-to-NAD in the order of 0.03-0.07 and NADPH-to-NADP 10-100 folds higher [50]. Moreover, since our experimental data on the cell contents in these single nucleotides suggest that their respective sums (ATP+ADP+AMP; NAD+NADH; NADP+NADPH) vary of lower amplitudes than the ratios during a batch culture; it has thus been decided to keep with using ratios, as we have recently used to describe another CHO cell line [36]. This approach has also been suggested by Dash et al. (2008) [51] to model metabolism and energetics in Skeletal Muscle cells. We have thus considered using these nucleotide ratios as the driving forces coordinating metabolic reactions. Moreover, the uptake rates of extracellular metabolites (glucose, glutamine, amino acids) are the dominant factors driving changes in the metabolic system. However, the Km values for most metabolite transporters are low relative to the extracellular concentration of the metabolites (Tables S6–S7) [52]. This suggests that the transport of a metabolite into a cell may not be mainly controlled by the transporter, but rather from the intracellular enzymatic reactions and regulation. The extracellular concentrations influence the dynamics of intracellular concentrations. Therefore, it is proposed in this work to model the consumption of extracellular metabolites through the intracellular enzyme-catalyzed reactions with related kinetic rate expressions as suggested by [53], where the concentration dependencies of the kinetic expressions are based on the corresponding extracellular metabolite concentration. In this work, additional regulatory functions, mainly in glycolysis (Figure 2), were introduced and evaluated, one by one, to either describe activation or inhibition of enzyme kinetics. The regulatory mechanisms involved in glycolysis are described as hexokinase inhibition by its product G6P (term I), phosphoglucose isomerase (term II) and phosphofructokinase inhibition (term III) respectively by PEP and G6P, activation of pyruvate kinase by F6P (term IV), as well as the inhibition of lactate dehydrogenase forward reaction (term V) were considered based on information derived from the literature [54]–[56]. Activation and inhibition mechanisms of the enzymatic reactions are expressed through negative and positive feedback and feedforward loops, modifying the Michaelis-Menten rate laws as illustrated in Figure 2 [57]. Finally, reaction reversibility has been taken into account, for those showing negative flux rate values during the course of a culture simulation: understanding that model simulations were not restricted in their signs. Thermodynamics aspects of biochemical kinetics were not considered in this work, because the model includes mainly lumped biochemical reactions.

### Model calibration

The final fully dynamic model includes 35 reactions and 46 variables. The kinetic formulations for the flux regulation are presented in Table S5. The model has 95 kinetic parameters, 48 affinity constants (Table S7), 42 maximum reaction rates (Table S8) and one parameter for each regulatory function (Table S7). Initial conditions for most of the variables were available from culture data (Table S6), while those remaining were taken from literature for similar conditions (*brenda-enzymes.info* and references therein [48]). The set of kinetic parameters previously determined for another CHO cell line in bioreactor cultures [36] was used as initial estimates, when described, and the new parameters were taken from literature for similar biological systems (*brenda-enzymes.info* and references therein [48]). The parameter estimation approach used is extensively discussed in a previous study [36]. Briefly, for each of the five cultures under investigation, a sensitivity analysis was performed for evaluating the influence of each parameter on the model output. In order to define their influence, parameters were systematically varied from their initial value comparing respective model output, defined as the weighted sum of squared residuals (WSSRES) between available experimental data (*X^mea^*) and simulated values (*X^sim^*) for each state variable *m* at time *k*, where the weight is the inverse of the variance of the experimental data for each state variable, *var_m_* ^−1^:
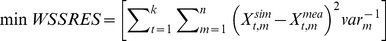



The sensitivity analysis procedure allowed to rank the parameters by their decreasing influence, and to remove parameters that were not contributing to model sensitivity from further optimization cycle, keeping them at their initial value. Optimal parameter values (for the sensitive ones) were then obtained by minimizing the normalized sum-squared errors using a *Least-squares* minimization function in MATLAB's Optimization Toolbox (The Mathworks, Inc., Natick, USA) for non-linear regression. Finally, 95% confidence intervals for both model sensitive parameters and model predictions were calculated using built-in MATLAB functions “*nlparci.m”* and *“* *nlpredci.m”,* respectively.

## Results

### Model structure fine-tuning and characterization

The model was first applied to parental cell line culture data obtained in shake flasks. Model performance assessment with cumate-induced and non-induced cultures of low-producer and high-producer clones is presented thereafter, and the details on model parameters calibration are shown as (Figures S1, S2, S3).

#### Describing the regulation of glycolysis ameliorates model simulations of experimental data

Biologically relevant scenarios (Figure 2) of enzyme regulation mechanisms, known to play a role in glycolysis robustness, were successively evaluated from model performance to simulate experimental data. For clarity reasons, only simulations for four significant model variables, such as cell density, glucose, ATP-to-ADP and NADH-to-NAD ratios, are shown here (Figure 2) for parental and the induced low- and high-producer cultures, and the remaining results can be found as (Figures S4, S5, S6, S7, S8, S9, S10). The last two model variables are markers of cell energetic and redox states, respectively [58]. Interestingly, one can observe that model simulations of cell growth agreed with experimental data in all regulation scenarios. However, the error between model simulation and experimental data for extracellular glucose and energetic nucleotides ratio shows to be high, when no regulation terms are included in the kinetic flux expressions. Hexokinase inhibition by G6P (term I) decreases the simulation error for extracellular glucose and redox nucleotides ratio, and to a lesser extent for energetic nucleotides ratio. Adding a term (II) to account for PGI inhibition by PEP further reduces the error between model simulations and experimental data, and this is particularly obvious for energetic nucleotide ratios. This suggests that variations in sugar phosphate cell concentrations, although of low magnitudes, may trigger the first two regulatory mechanisms to control glycolytic fluxes. To verify whether incorporating other regulatory terms into rate expressions significantly influences simulation results, a formulation with all main regulatory steps was also tested. The last formulation also shows to allow simulating experimental data almost similarly to the case when the first two terms are considered. This may suggest that not all the regulatory terms are solicited within experimental conditions in this study. Therefore, because of a higher performance level as well as a lower formulation complexity, a kinetic formulation including the first and the second regulatory terms was used in the remaining of this study. Considering extracellular metabolites (Figures S4,S6,S8) the model is far from predicting experimental data when no regulatory terms are considered. However, model simulations are closer to experimental data when only adding the first (term I) and the second (term II) regulatory terms, respectively hexokinase feedback inhibition by its product, G6P, and phosphoglucose isomerase inhibition by PEP. Interestingly, our experimental data set accounts for a wide diversity of metabolites such as by-products (lactate, NH_4_^+^, glutamate), amino acids (alanine, glutamine, serine, aspartate, and amino acid pools to TCA cycle), sugar phosphates of glycolysis (G6P and PEP) as well as glucose, cell density and energetic nucleotides, which are all well simulated by the model implemented with regulation terms I and II. Similar observations can be drawn for scenarios of intracellular organic acids such as PYR and SUC. In addition, model simulations corresponded more closely to experimental data for organic acids such as AKG and MAL in induced low- and high-producer cultures, but to a lesser extent to no clear effect for other amino acids and AMP (Figures S5,S7,S9). Finally, the same behavior can be observed for the cell specific oxygen consumption rate (qO_2_), for which simulations were closer to experimental data comparing to the case with no regulation (Figures S4,S6,S8).

#### A limited subset of model parameters drives the in silico cell behavior

A sensitivity analysis was performed on the resulting model, aiming to identify the most critical parameters. Values of model parameters were changed from −85 to +300%, one at a time, from their optimal value, and the normalized sum-squared differences (*WSSRES)* were calculated as previously described. Resulting *WSSRES* values were then further normalized to that obtained for original optimal parameter values (i.e. 0% change). Parameters showing a deviation of ±15% and higher were considered sensitive; a colormap (Figure S1) was drawn to illustrate the extent to which normalized *WSSRES* values vary from that of the optimal value (i.e. minimal simulation error). The model reveals to be primarily sensitive to parameters of glycolysis, TCA cycle and energetic reactions, amino acids catabolism pathways, partially to glutaminolysis, and to a lesser extent to the pentose phosphate pathway. The specific glucose uptake rate (ν*_maxHK_*) and other parameters of glycolysis (*ν_maxPGI_, ν_maxPK_, ν_maxf LDH_* and *ν_maxr LDH_*) show to strongly affect simulation error. Moreover, maximum reaction rates for three enzymes in TCA cycle (*ν_maxAKGDH_*, *ν_maxCS_,* and *ν_maxMLD_*), and for the reaction connecting glycolysis to TCA cycle (*ν_maxPDH_, ν_maxME_,* and *ν_maxPC_*), also reveal to be significant. The model is also highly sensitive to three reactions related to glutaminolysis (*ν_maxfGLNS_, ν_maxrGLNS_*, *ν_maxfGLDH_, ν_maxrGLDH_, ν_maxfAlaTA_,* and *ν_maxASX_*), and to a lesse extent to two parameters related to the pentose phosphate pathway oxidative branch (*ν_maxG6PDH_,* and *ν_maxEP_*). The model shows a high sensitivity to energetic reactions, represented here by parameters related to non-specifically described ATP (*ν_maxATPase_*) and NADH consuming reactions (*ν_maxresp_*). Furthermore, the maximum specific growth rate (*ν_maxgrowth_*) also strongly influences the simulation error. Finally, parameters related to amino acids catabolism (*ν_maxrAlaTA_, ν_maxSAL_, ν_maxfASTA_, ν_maxHISARGTA_,* and *ν_maxLYSILELEUHISVALTYRTA_*) also demonstrate to be influential. There are therefore a high number of non-influential parameters with 65 out of 95. This lack of sensitivity may partially come from the experimental space used to calibrate and to challenge the model. Although these non-sensitive parameters are biologically relevant, describing existing active pathways and enzymatic reactions, they may require expanded experimental culture conditions to be solicited, as we proposed in a recent work [59]. For space limitation only, sensitivity results for parental culture are shown while the other cultures exhibited almost the same results. Therefore, the model was kept as is at this point because actual non-sensitive parameters may become sensitive and thus be useful in a future study exploring outside the actual experimental space.

#### A limited subset of measured variables contribute to the overall model sensitivity

The specific contribution of each measured variable to the overall model sensitivity was also investigated. Among measured variables, cell density, extracellular glucose, glutamine, lactate, ammonia, ASX (ASN+ASP), amino acids pool to glutamate and nucleotide ratios showed a high sensitivity level compared to pyruvate and succinate (Figure S2). Not surprisingly, energetic nucleotide ratios exhibited the highest sensitivity as it is affected by multiple reactions in various parts of the metabolic network, through their regulatory role. Interestingly, parameters with a relatively high global sensitivity on model overall output may not systematically impact on all variables simulated. Here again, results are conditioned by the experimental space studied. It may thus suggest that experimental intracellular concentrations have never reached threshold levels, above or below which a higher impact could have been observed. The whole procedure of model parameters calibration has then been performed on the cumate-inducible cell lines, induced and non-induced. For space limitation and clarity reasons, only final calibrated results are shown and discussed in the following sections (see Tables S7 and S8 for parameters values).

### Assessment of the *in silico* platform performance

#### The model describes intra- and extracellular metabolites concentration profiles and growth kinetics

**Figure 3:**
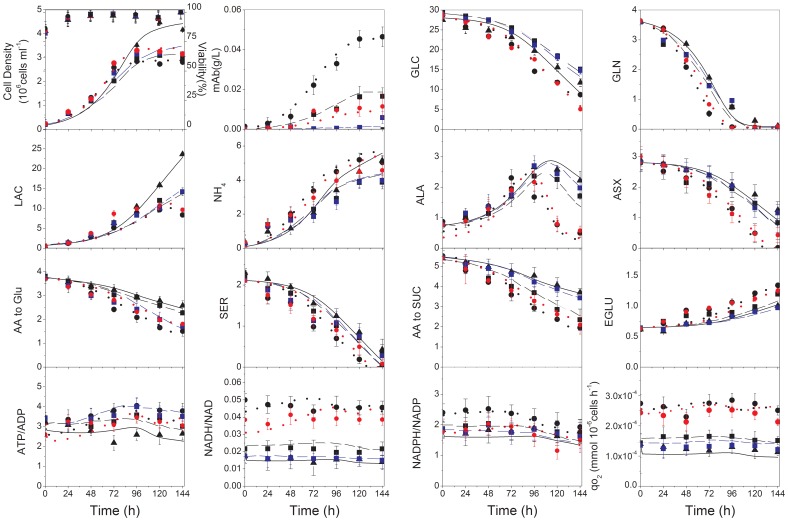
Simulated and experimental data for parental and induced/non-induced cell lines. Parental (experimental data: black triangles, simulated data: solid black line), induced low-producer (experimental data: black squares, simulated data: dashed black line), non-induced low producer (experimental data: blue squares, simulated data: dashed blue line), induced high-producer (experimental data: black circles, simulated data: dotted black line), and non-induced high-producer (experimental data: red circles, simulated data: dotted red line).

The kinetic expression for the cell specific growth rate, as multiplicative Michaelis-Menten kinetics for precursors of cell building blocks, was able to simulate the viable cell concentration profile in all CHO cells cultures under study (Figure 3). High- and low-producing clones exhibit almost similar growth profiles, reaching maximum viable cell densities of ∼3.5×10^6^ cells mL^−1^, while parental cell line reached slightly higher maximum viable cell density (∼4.5×10^6^ cells mL^−1^). The model also simulates extracellular metabolites profile with time, although significant differences in behavior are observed between the parental, low- and especially high- producer clones (Figure 3). Interestingly, differences between induced and non-induced cultures of the same clone are non-significant. Irrespective of the clone or induction state, all cultures were not glucose-limited (>5–15 mM at culture harvest), with the higher consumption in high-producer clone cultures. Parental and low-producer cultures exhibit similar glutamine profiles, and a faster depletion is again observed for the high-producer clone. Growth cessation coincided with the depletion of glutamine. Interestingly, unlike glucose, lactate concentration profile differs among clones but the model structure is able to simulate each case. Lactate is produced all along cultures but, however, the high-producer clone seems to start consuming lactate at glutamine depletion, suggesting the coupling of these phenomena as suggested by Zagari et al. (2013) [60]. Ammonia production was almost similar in parental and low-producer cultures, reaching a final concentration of approximately ∼4 mM whereas it was ∼5 mM in the case of the high-producer clone. Similarly to lactate, the high-producer clone seems to start consuming ammonia following glutamine depletion. Differences in extracellular metabolites profiles are significantly related to amino acids metabolism (see Table S8 for statistical analysis). Globally, all amino acids except alanine and glutamate are consumed and the consumption/production rates are greater in the case of high-producer clone (Figure 3). Glutamate concentration constantly increases in all culture media, and alanine is also constantly produced during exponential phase but consumed thereafter (from ∼96 h), with a more pronounced decrease in the high-producer clone. In that culture, alanine may have compensated for the lack of glutamine, once the latter was depleted. Beside alanine, extracellular concentrations in (ASN+ASP) and SER in the high-producer reached depletion. These amino acids are expected to contribute to pyruvate synthesis. Although a higher consumption of grouped amino acids channeled through succinate and glutamate can be identified in the high-producer culture, there is no depletion observed. Interestingly, most intracellular metabolites show constant and similar levels between cultures except for G6P and PEP with an increasing trend after exponential phase (Figure S10).

#### Model simulates CHO cells clonal variations in energetic state

As previously mentioned for the parental cell line, the cell energetic state represented by ATP-to-ADP, NADH-to-NAD ratios, and the cell specific oxygen consumption rate (*qO_2_*) are well simulated for all cultures (Figure 3). Although the cell specific oxygen consumption rate is generally greater for the high-producer clone, the ATP-to-ADP ratio, a marker of respiration and energy consumption, showed relatively stable and similar values in all cultures. NADPH-to-NAPD was also substantially similar and stable in exponential phase in all cell lines, with a slight decrease after exponential phase, which suggests the down-regulation of NADPH production. Finally, the NADH-to-NAD ratio, which is a marker of TCA cycle activity, was considerably higher in high-producer clone than in parental and low-producer cell lines, indicating a sustained up-regulated TCA activity, as discussed in the next sections.

#### Clonal variation in physiology can be inferred from a limited set of model kinetic parameters

In order to further evaluate parameters adjustment attributed to clonal variation, the associated p-values for each pair of estimates (control vs. either induced low-producer or high-producer cultures) were calculated (Table S8). In low- and high-producer clones, only two and four parameters, respectively, were statistically different from those for parental to allow the model to simulate the effect of cumate induction. Briefly, in the case of the induced low-producer clone, main differences can be observed for parameters related to glycolysis (*ν_maxrLDH_*), and ATP consumption reactions, which are lumped as ATPase proton pumps requirements (*ν_maxATPase_*). However, in the case of the induced high-producer clone, *ν_maxr LDH_* and *ν_maxATPase_* are both significantly changed in addition to one parameter related to glutaminolysis (*ν_maxfASTA_*) and one related to the reactions connecting glycolysis to TCA cycle (*ν_maxPDH_*). The high-producer clone thus resulted in a significantly different *in silico* behavior compared to the parental cell line and, to a lesser extent, to the low-producer clone (Figure S3 and Tables S8) regarding the simulations that are however in agreement with experimental data. Interestingly, the high-producer clone only requires the adjustment of four parameters values from those of the parental for the simulations to cope with experimental data.

#### The model simulates mAb production

The dynamics of mAb production, modeled as multiplicative Michaelis-Menten kinetics of amino acids, resulted in the simulation of mAb titers (Figure 3) both in low- and high-producer clones. The model thus shows to simulate experimental data in induced cultures while it simulates the production resulting from the leaky expression of the inducible system.

## Discussion

### The kinetic-metabolic model is a reliable *in silico* tool to assess CHO cells clonal variations

Induction of recombinant proteins in microbial cell platforms has been shown to cause an increased energetic demand in support to a metabolic burden [61], [62]. Unlike microbial cells, the links between cell metabolic load and protein productivity in engineered mammalian cells has yet to be tackled, although some progress has been accomplished with the help of ^13^C-labeling [29]. In this work, an inducible system with low- and high-producer clones have been selected in order to study an induction effect on CHO cell metabolic behavior and load. Towards this goal, the use of an *in silico* platform, made of a kinetic-metabolic model, confers a unique capacity to explore mAb CHO producing cells beyond experimental observations onto which the model has been anchored *a priori*. Therefore, the remaining discussion will be based on the results derived from the developed *in silico* platform.

### Clone to clone variations yield more significant metabolic changes than recombinant protein expression

**Figure 4:**
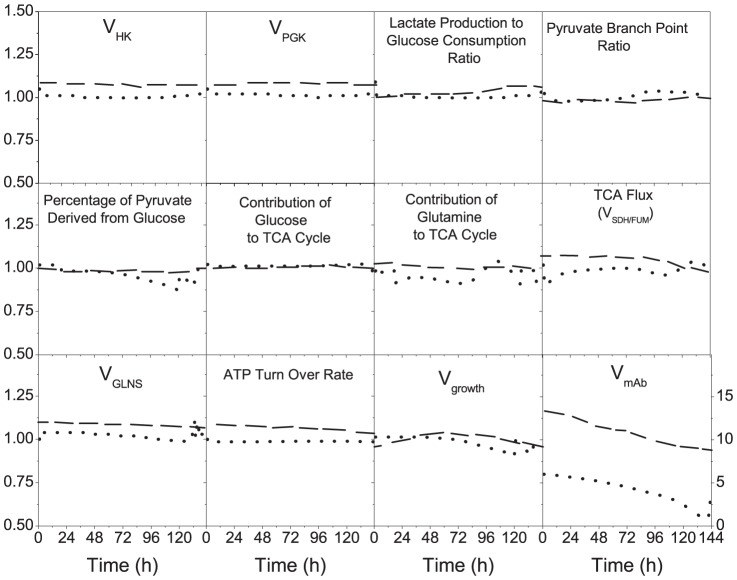
Comparison of metabolic fluxes and ratios. Specific glucose uptake rate (*ν(HK*)), glycolytic flux*(ν(PK)),* lactate production-to-glucose consumption ratio (*(νf(LDH)-νr(LDH))/ν(HK)),* pyruvate branch point as the ratio of the pyruvate influx through TCA cycle divided by the total flux into pyruvate pool ((*v(PDH)+ ν(PC)/(ν(PK)+ν(SDH)+ν(ME)+ν(AlaTA*)), when the last two fluxes positively fed pyruvare, percentage of pyruvate derived from glucose (*v(PK/(ν(PK)+ν(SAL)+ν(ML-PC)+ν(AlaTA*)), Contribution of glucose to TCA cycle as the ratio of pyruvate influx to TCA cycle via *ν(PDH)*, considering most of the *ν(PDH)* has been originated from *ν(PK),* to the total flux channeled through TCA cycle via its intermediates (*v(PDH)/(ν(PDH)+ ν(ASTA)+ ν(GLDH)+ ν(LYSILELEUVALTYRTA)+ν(PC)),* Contribution of glutamine to TCA cycle as the ratio of glutamate influx to TCA cycle via *ν(GLDH)* to the total flux channeled through TCA cycle via its intermediates (*v(GLDH)/(ν(PDH)+ν(ASTA)+ν(GLDH)+ν(LYSILELEUVALTYRTA)+ν(PC))),* Contribution of other amino acids to TCA cycle ((*v(LYSILELEUVALTYRTA)+ν(ASTA))/(ν(PDH)+ν(ASTA)+ν(GLDH)+ν(LYSILELEUVALTYRTA)+ν(PC))),* TCA cycle flux (*ν(SDH/FUM)*), specific glutamine uptake rate (*νf(GLNS)-νr(GLNS*)), ATP turnover rate (*ν(PGK)+ν(PK*)+v(SCOAS)+νr(GlnT)+ νf(CK)+vr(AK)+2P/O ratio*ν(resp)), Specific growth rate *ν(growth)*, and specific production rate *ν(mAb),*between induced and non-induced low-producer (dashed line) and high-producer (dotted line) cell lines. The values are defined as the ratio of specific metabolic fluxes (mmol (10 ^6^cells)^−1^ h^−1^) or ratio in induced cultures to that in the non-induced control cultures at each time point.

In the previous sections, we reported large differences in behavior between the low- and the high-producer clones, comparing non-induced and induced cells (Figure 3). However, it is of interest to evaluate the source of these differences. The contribution of mAb production is estimated to account for atmost 5% of total carbon uptake by the cells, as previously observed [29], even for the high-producer clone, and one can expect the *de novo* metabolic load associated to the recombinant proteins to be low compared to the intrinsic one related to endogeneous protein synthesis. This estimate is calculated taking the carbon mass in 1 mol of mAb to the augmentation of the total mass of carbon from cellular growth, considering a specific productivity of ∼1×10^−6^ mmol 10^−6^ cells h^−1^ and a specific growth rate of ∼0.04 h^−1^, and assuming a dry cell weight of 350 pg cell^−1^, a cellular molecular weight of 150 g mol^−1^ and the reported elemental formulas for both biomass and mAb [34]. Therefore, the production capacity is not thought to be limited at the anabolic level, but rather at the protein processing stage (assembly and the folding) [63]. We then used the model to evaluate the effect of cumate induction on the metabolic load, and similar intracellular flux distribution, metabolic fluxes and ratios were found when normalized to their time-corresponding values in non-induced controls (Figure 4). Only metabolic fluxes and ratios of major metabolic networks such as glycolysis and TCA are shown. Interestingly, most normalized values of metabolic fluxes and ratios are close to 1 for the high-producer clone, while the low-producer clone exhibits deviations from 0 to 10% for a series of fluxes with +10% for *V_HK_*, *V_PGK_*, *V_GLNS_*, TCA flux and ATP turnover rate. In the case of the high-producer clone, the contribution of glutamine to TCA cycle is lower in the induced culture. Indeed, the higher deviation between induced and non-induced cells is observed comparing the mAb specific production rates with time, with production rates that are 13 to 8 times higher in the low-producer clone and 5 to 1 times higher in the high-producer clone. This higher deviation level in the low-producer clone looks surprising but it can be attributed to a higher leakage level of the cumate gene switch in the high-producer clone in the absence of cumate induction (Figure 3). Our results, both experimental and from simulations, thus suggest that within our experimental conditions, differences in metabolic time profile caused by clonal variation [24] exceeded that induced by recombinant protein expression [61], [62].

### High producer clone selection favors metabolically efficient cell population subsets

#### The high-producer clone shows a more efficient lactate metabolism

**Figure 5:**
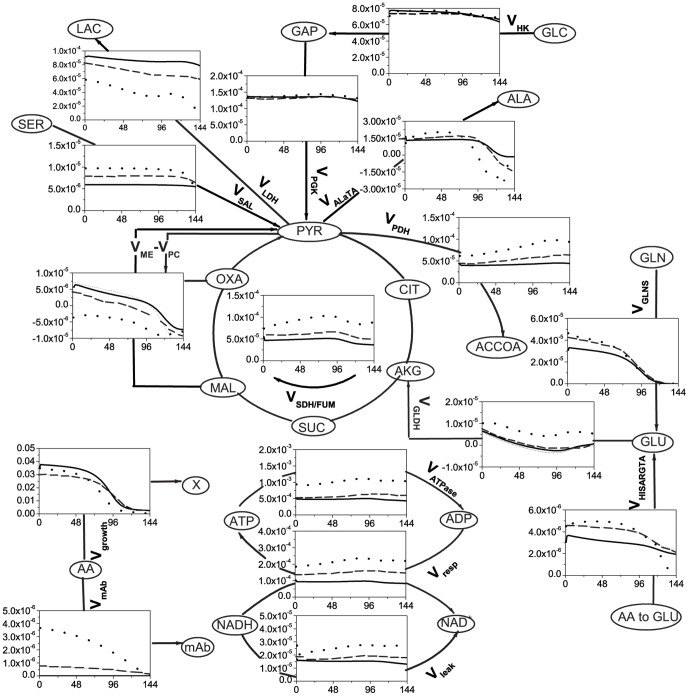
Selected metabolic fluxes of parental and induced low- and high-producer cell lines. Parental (solid line), induced low-producer (dashed line), and induced high-producer (dotted line). The fluxes (*y*-axis) are given in mmol (10^6^ cells)^−1^ h^−1^ and the time (*x*-axis) in hours. Negative values indicate fluxes in the opposite direction of the arrow.

**Figure 6:**
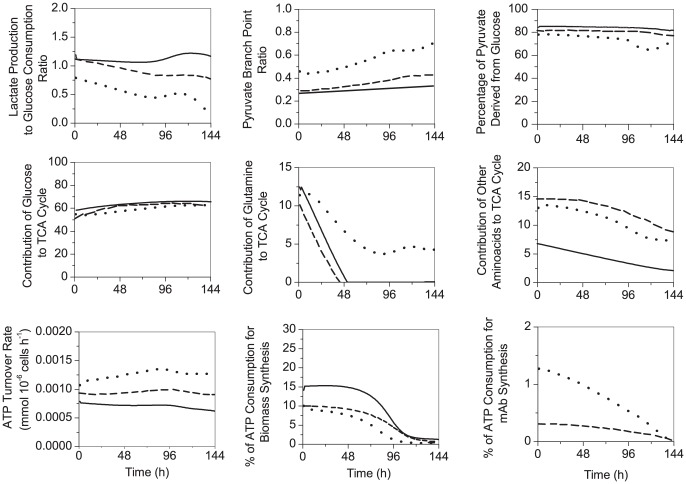
Comparison of metabolic ratios. Lactate production-to-glucose consumption ratio (*(νf(LDH)-νr(LDH))/ν(HK)),* pyruvate branch point as the ratio of the pyruvate influx through TCA cycle divided by the total flux into pyruvate pool (*v(PDH/(ν(PK)+ν(SAL)+ν(ML-PC)+ν(AlaTA*)), when the last two fluxes positively fed pyruvare, percentage of pyruvate derived from glucose ((*v(PK)+ ν(PC))/(ν(PK)+ν(SAL)+ν(ML)+ν(AlaTA*)), Contribution of glucose to TCA cycle as the ratio of pyruvate influx to TCA cycle via *ν(PDH)*, considering most of the *ν(PDH)* has been originated from *ν(PK),* to the total flux channeled through TCA cycle via its intermediates (*v(PDH)/(ν(PDH)+ ν(ASTA)+ ν(GLDH)+ ν(LYSILELEUVALTYRTA)+ν(PC)),* Contribution of glutamine to TCA cycle as the ratio of glutamate influx to TCA cycle either via *ν(GLDH)* to the total flux channeled through TCA cycle via its intermediates (*v(GLDH)/(ν(PDH)+ν(ASTA)+ν(GLDH)+ν(LYSILELEUVALTYRTA)+ν(PC))),* contribution of other amino acids to TCA cycle ((*v(LYSILELEUVALTYRTA)+ν(ASTA))/(ν(PDH)+ν(ASTA)+ν(GLDH)+ν(LYSILELEUVALTYRTA)+ν(PC)))*, ATP turnover rate (*ν(PGK)+ν(PK*)+v(SCOAS)+νr(GlnT)+ νf(CK)+vr(AK)+2P/O ratio*ν(resp)), percentage of ATP consumption for biomass synethesis(0.00043*3.78*ν(growth)/(*v(LYSILELEUVALTYRTA)+ν(ASTA))/(ν(PDH)+ν(ASTA)+ν(GLDH)+ν(LYSILELEUVALTYRTA)+ν(PC)*), and percentage of ATP consumption for antibodysynthesis(4*ν(mAb)/(*v(LYSILELEUVALTYRTA)+ν(ASTA))/(ν(PDH)+ν(ASTA)+ν(GLDH)+ν(LYSILELEUVALTYRTA)+ν(PC)*),between parental (solid line), induced low-producer (dashed line) and induced high-producer (dotted line) cell lines.

Model simulations (Figure 4) suggest that the distinct metabolism of the high-producer clone favors mAb production irrespective of cumate induction. Induced and non-induced high-producer show similar glycolytic rates (*V_HK_*, *V_PGK_*), glutamine metabolism (*V_GLNS_*) and ATP turnover rate for the whole culture duration. However, interestingly, although hexokinase and phosphoglucose isomerase activities are both not affected by cumate induction, lactate dehydrogenase activity shows the lowest values for the high-producer clone, and to a lesser extent for the low-producer clone (Figure 5). Lactate production rate for the high-producer clone is lower (−46% at mid exponential phase and −56% at the end of exponential phase) than that for parental (Figure 4, Table 1). Therefore, although an overflow of glycolytic flux to lactate has been extensively reported under non-limiting glucose conditions [64], irrespective to recombinant protein expression, the high-producer CHO cell clone seems to maintain a more efficient metabolic state; a result that is also supported form simulated lower values of lactate production rate-to-glucose consumption rate ratio (Figure 6). While a quasi-constant ratio value is maintained for the parental clone, the low-producer also exhibits a decreasing trend, but to a lower extent than for the high-producer. With ratios lower than 1, while literature usually reports a 1-2 range, one can clearly conclude of a respiratory metabolism, coupled to a high feeding rate of intermediates to anabolic reactions.

**Table 1:** Comparison of metabolic fluxes in parental, induced low-producing and induced high-producing cell lines.

At 48 h							
Metabolic Flux	parental		Low-producing		High-producing		t test
	Value	Interval	Value	Interval	Value	Interval	
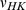	7.67E-5	[6.33E-5,9.03E-5]	7.32E-5	[6.11E-5,8.52E-5]	7.66E-5	[6.14E-5,9.18E-5]	–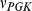	1.35E-4	[8.19E-5,1.88E-4]	1.31E-4	[8.78E-5,1.74E-4]	1.38E-4	[1.08E-4,3E-4]	–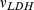	8.74E-5	[7.13E-5,1.04E-4]	7.36E-5	[5.68E-5,9.09E-5]	4.49E-5	[2.68E-5,6.30E-5]	p<0.1^+^
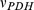	4.04E-5	[2.64E-5,5.45E-5]	4.86E-5	[3.65E-5,6.08E-5]	7.06E-5	[5.60E-5,8.52E-5]	p<0.1^+^
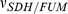	4.90E-5	[3.23E-5,6.57E-5]	6.16E-5	[4.68E-5,7.63E-5]	9.35E-5	[7.65E-5,1.11E-4]	p<0.1^+^
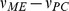	3.70E-6	[1.73E-6,5.67E-6]	4.72E-6	[2.75E-6,6.69E-6]	-3.57E-6	[-1.86E-6,-5.27E-6]	p<0.1^+^
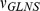	2.91E-5	[2.05E-5,3.80E-5]	3.89E-5	[2.69E-5,3.89E-5]	3.81E-5	[2.74E-5,4.87E-5]	–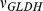	5.27E-7	[2.43E-7,8.11E-7]	3.23E-7	[1.19E-7,5.27E-7]	6.73E-6	[3.29E-6,1.02E-5]	p<0.1^+^
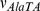	1.36E-5	[9.46E-6,1.77E-5]	1.61E-5	[1.20E-5,2.02E-5]	2.02E-5	[1.52E-5,2.53E-5]	–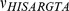	3.16E-6	[2.14E-6,4.25E-6]	4.38E-6	[3.14E-6,5.62E-6]	4.99E-6	[3.45E-6,6.53E-6]	–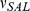	5.95E-6	[5.67E-6,6.23E-6]	7.95E-6	[4.37E-6,1.15E-5]	9.71E-6	[6.12E-6,1.33E-5]	–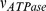	4.78E-4	[2.13E-4,7.43E-4]	5.51E-4	[2.86E-4,8.16E-4]	9.93E-4	[4.27E-4,1.56E-3]	–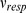	9.32E-5	[4.99E-5,1.36E-4]	1,44E-4	[1.09E-4,1.79E-4]	2.10E-4	[1.68E-4,2.53E-4]	–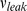	1.52E-5	[9.06E-6,2.13E-5]	1.75E-5	[8.16E-6,2.68E-5]	2.47E-5	[1.16E-5,3.77E-5]	–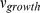	0.035	[0.031,0.038]	0.028	[0.025,0.031]	0.031	[0.026,0.035]	–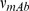	–	–	6.43E-7	[5.43E-7,7.43E-7]	3.11E-6	[2.21E-6,9.00E-7]	–

#### *Anaplerosis/cataplerosis* requirements allows for different flux distribution around pyruvate node in the high-producer clone

Lower values for the lactate production rate-to-glucose consumption rate ratio were concomitant to higher fluxes through pyruvate dehydrogenase in the high-producer clone. Pyruvate dehydrogenase activity remains almost constant in parental, while it increases of 75% in high-producer and of 45% in low-producer at mid exponential phase (Figure 5, Table 1). In addition, both pyruvate carboxylase and malic enzyme show non-zero fluxes in all clones (Figure 5). Our values agree, in order of magnitude, with non-zero values that have been recently estimated for CHO cells using isotopic tracers technique [27]. These anaplerotic/cataplerotic reactions are known to be important for the replenishment of TCA-cycle intermediates [65]. Unlike parental and low-producer, the balance between these two fluxes favors the formation of oxaloacetate from the beginning of the culture, which implies a higher activity of pyruvate carboxylase and a lower activity of malic enzymes in high-producer clone. Higher efflux of malate out of TCA cycle implies a higher rate for its conversion to pyruvate, and finally to lactate in parental clone. The latter agrees with higher values of lactate production rate, both observed experimentally and from simulations (Figure 5, Table 1). In addition, slightly higher values of the NADPH-to-NADP ratio suggest a greater contribution of malic enzyme in both lactate and NADPH production in parental cell line. Moreover, a higher pyruvate carboxylase activity in the high-producer may decrease the available pyruvate pool, which could *in fine* reduce lactate formation rate. The anaplerotic flux through glutamate dehydrogenase stay moderate in all three cultures, suggesting that acetyl coenzyme A derived from pyruvate is the predominant intermediate fuelling the TCA cycle. This result is supported by a high glucose contribution to TCA cycle (50–60%) (Figure 6, Table 2). A higher portion of pyruvate directed to TCA leads to higher values of the pyruvate branch point ratio (+75%) estimated in high-producer, and in low-producer to a lesser extent (Figure 6, Table 2). While a large fraction of pyruvate enters the TCA cycle (55 to 75%), only 15–35% is converted into lactate in the high-producer clone. A lower value of lactate production rate-to-glucose consumption rate has been associated to the over-expression of pyruvate dehydrogenase in other animal cells [66], [67]. The fraction of pyruvate entering the TCA is noticeably higher compared to values previously reported for other CHO cell lines [68], [69], but they are in agreement with recent reports on low values of lactate production [70], or even showing a net lactate consumption [27]. The pyruvate branch point ratio shows an increasing trend in the high-producer clone, with a more active TCA cycle along culture time. Independently of the clone, the flux distribution around the pyruvate branch point suggests that a high proportion of pyruvate is derived directly from glycolysis (∼80%) (Figure 6, Table 2), while the remaining 20% may mainly originate from malic enzyme activity through the efflux of malate from TCA cycle, and amino acids catabolism. The estimated flux from malate to pyruvate is high at the beginning of the culture, but this flux drops and stays at a low value as culture is progressing (Figure 5); a behavior that has also been reported [71].

**Table 2:** Comparison of metabolic ratios in parental, induced low-producing and induced high-producing cell lines.

At 48 h							
Metabolic Ratio	parental		Low-producing		High-producing		t test
	Value	Interval	Value	Interval	Value	Interval	
1	1.07	[0.75,1.39]	0.97	[0.69,1.25]	0.58	[0.43,0.73]	p<0.1^+^
2	0.26	[0.15,0.36]	0.33	[0.14,0.51]	0.54	[0.37,0.70]	p<0.1^+^
3	84.9	[71,98]	85.42	[68,101]	76.75	[62,91]	–
4	65.03	[50,79]	61.95	[47], [76]	57.05	[41], [72]	–
5	1.21	[0.83,1.58]	–	–	7	[5.23,8.76]	p<0.1^+^
6	4.89	[2.80,6.97]	14.35	[10.52,18.17]	12.54	[9.45,15.62]	–
7	7.20E-4	[3.69E-4,1.07E-3]	9.36E-4	[4.85E-4,1.38E-3]	1.24E-3	[7.28E-4,1.75E-3]	–
8	7.95	[5.57,10.33]	4.91	[3.03,6.79]	3.91	[2.53,13.81]	–
9	–	–	0.28	[0.26,0.31]	1.02	[0.82,1.22]	p<0.1* ^+^

At 74 h							
Metabolic Ratio	parental		Low-producing		High-producing		t test
	Value	Interval	Value	Interval	Value	Interval	
1	1.06	[0.65,1.46]	0.88	[0.56,1.20]	0.47	[0.29,0.65]	p<0.1^+^
2	0.29	[0.16,0.41]	0.35	[0.14,0.55]	0.55	[0.33,0.76]	p<0.1^+^
3	84.55	[70,98]	84.68	[71,98]	75.44	[62,88]	–
4	66.02	[50,81]	63.02	[47,78]	59.36	[41], [76]	–
5	–	–	–	–	4.47	[2.97,5.96]	p<0.1^+^
6	4.15	[1.92,6.37]	13.44	[10.82,16.05]	11.47	[9.40,13.53]	–
7	7.19E-4	[4.00E-4,1.00E-3]	9.72E-4	[5.21E-4,1.41E-3]	1.32E-3	[7.43E-4,1.89E-3]	–
8	6.86	[3.95,9.77]	4.19	[2.28,6.11]	2.72	[1.81,3.63]	–
9	–	–	0.24	[0.14,0.33]	0.81	[0.69,0.91]	p<0.1* ^+^

The numbers in the left column correspond to the following metabolic ratios: 1: lactate production- to-glucose consumption, 2: Pyruvate branch point ratio, 3: percentage of pyruvate derived from glucose, 4: contribution of glucose to TCA cycle, 5: contribution of glutamine to TCA cycle, 6: contribution of other amino acids to TCA cycle, 7: specific ATP production rate (mmol (106 cells)-1h-1)), 8: percentage of ATP consumption for biomass synthesis, 9: percentage of ATP consumption for mAb synthesis.

#### Ammonia accumulation impairs the contribution of glutamine-metabolism to TCA cycle activity in all clones except in high-producer

Although glutamine has been shown to be the major amino acid catabolized in the TCA cycle [29], [45], its contribution to TCA decreases with respect to time to values close to zero at ∼48 h for the parental and the low-producer clones only, while it reduces to ∼4.5% for the high-producer clone at 72 h for then remaining quasi-constant until the end of the culture (Figures 5,6). It even appears that the direction of the glutamate dehydrogenase flux is reversing from a α-ketoglutarate-producing (positive flux) to a glutamate-producing (negative flux) reaction in parental and low-producing cultures. This result suggests that the CHO cell lines under study may redirect the flux through glutamate dehydrogenase when the ammonia concentration increases, since glutamate dehydrogenase provides an alternative for the uptake (i.e. detoxification) of NH_4_^+^. A reversed flux has been reported in literature from a medium concentration threshold of 10 mM NH4^+^ [72]. However from our experimental data, it seems that the reverse direction of the glutamate dehydrogenase flux is favored at extracellular ammonia econcentrations even below that threshold value. The fact that NH_4_^+^ is staying at concentrations within the order of magnitude of the Michaelis affinity constant of glutamate dehydrogenase for NH_4_^+^ (Km_NH4_) (0.5 to 3.2 mM) may partially explain the low level for the direct contribution of glutamine to TCA cycle via glutamate dehydrogenase. Interestingly, alanine, which is constantly produced during cell growth, is then consumed after glutamine depletion, and at higher rates for the high-producer, and to a lower extent for the low-producer. The alanine aminotransferase flux even shows to turn negative (Figure 5). This also suggests that under low glutamine concentration, α-ketoglutarate is re-channeled to the TCA cycle through aminotransferase, thus maintaining TCA cycle activity. The combined catabolism of all other amino acids represents ∼7% (parental) to ∼15% (low- and high-producers) of the total carbon metabolized through the TCA cycle (Figure 6), entering as either succinate or oxaloacetate. Therefore, the combined catabolism of all other amino acids represented a small but non-negligible fraction of the total carbon entering the TCA cycle. However, this contribution decreases of ∼5% with time in the three cultures. The slow increasing contribution of glycolysis (expressed as glucose contribution) to TCA cycle suggests that extracellular glutamine and other amino acids may contribute less with time as they are getting depleted from the culture medium. Further analyzing of both lactate and glutamine profiles reveals that the high-producer clone switches to lactate consumption toward the end of the culture from glutamine depletion (Figure 5). Once glutamine is depleted, lactate is readily consumed presumably to compensate for the reduced glutamine entry to TCA cycle. This also shows that generally, a low contribution of glutamine to TCA cycle may result in a lower net lactate production rate, resulting from a metabolic switch while glucose is still non-limiting, as observed in the high-producer clone and to a lesser extent in the low-producer clone.

#### The high-producer clone showed enhanced TCA cycle activity and ATP turnover rate

Greater pyruvate dehydrogenase and pyruvate carboxylase fluxes provide higher TCA cycle activity in the high-producer compared to the low-producer clone, and to the parental. Concurrently, the high-producer clone exhibits high values of NADH-to-NAD ratio (Figure 3). In agreement with this result, high-producer CHO cells were reported to have higher levels of intracellular NADH when compared to low-level producers [25]. Hence, a higher NADH-to-NAD ratio combined to higher TCA cycle fluxes in the high-producer suggests both active glycolysis and oxidative phosphorylation, meaning an intense production of intermediates as well as of energy. This result is in agreement with a higher ATP turnover rate simulated in this work and as reported in literature [73]. Indeed, cell respiration determined experimentally is well simulated by the model. Interestingly, the analysis of cell respiration rate and oxidative phosphorylation activity (Figure 3) reveals that ∼15% of the total oxygen uptake rate is not devoted to ATP-producing purposes but may be consumed through the proton leak phenomenon in the mitochondria. However, this is still speculative given the large confidence intervals associated with the flux representing ATP production, but it is in agreement with values reported in literature (10%–13%) [74]. Although high-producer cultures undergo metabolism with a high energy yield, the specific cell concentration in ATP stays constant (data not shown), whereas the ATP-to-ADP ratio is similar to that observed in the parental and low-producer cultures (Figure 3). This may be due to a higher mitochondrial proton leak in addition to a higher ATP consumption rate by the maintenance processes, which are lumped as ATPase flux in the model. Overall, simulations suggest that biomass synthesis only requires a minor part of ATP production with ∼15% (parental), ∼10% (low-producer) and ∼7% (high-producer) (Figure 6, Table 2). Recombinant protein synthesis is simulated to consume less than 2% of the ATP production rate, for the high- and the low-producer clones, and regularly decrease until the end of the culture (Figure 6, Table 2). The major portion of ATP production goes into maintaining catabolic and anabolic reactions, such as endogenous protein synthesis (80%–90%). Thus, as also suggested by Link et al. (2004) [75] the rate of oxidative phosphorylation, and consequently ATP production rate, may positively affect cell specific productivity but unlike bacterial cells [61], no direct correlation has been established yet linking the ATP turnover rate and the recombinant protein productivity in CHO cells. A higher ATP production rate may thus favor a better coordination of cellular functions, including enabling a better processing of endogenous proteins as well as of a recombinant protein, *in extensio*. This result correlates with our observation that ATP production rate is similarly elevated in both induced and non-induced high-producer cultures, i.e. independently of the recombinant mAb production rate. Taken together, it seems both from our experimental data and model simulations, that the higher productivity level of the high-producer clone in recombinant mAb is a consequence of its higher global metabolic activity. This up-regulation of central carbon metabolism was not a cause or a consequence of increased protein production load on cell metabolism but a clonal variation effect since the same result was also observed in non-induced cultures. Similar findings were reported in Chong et al. (2012) [76]. Therefore, our findings suggest that the major criterion for a successful clonal selection relies on the identification of clones showing a high metabolic efficiency and activity.

## Conclusions

This work on the characterization of different CHO mAb cell clones and their parental cell line, brings a wide set of experimental data for extra- and intracellular metabolites concentrations that were used to develop a descriptive and predictive kinetic-metabolic model. The *in silico* platform presented here enabled to better describe and quantify the metabolic differences resulting from CHO cells clonal variability. Such platform represents a valuable tool for cell line selection, as well as bioprocess development, but it may have also interesting applications in biomedical and medical applications. *In fine*, although this study is based on a large amount of experimental data for both culture media and cell content, including thermodynamic considerations on.

## Supporting Information

Figure S1 **Sensitivity analysis on model parameters for parental cell line culture.** The colormap represents the normalized sum of squared difference between model simulations and experimental data, when the parameter (row) is changed from −85% to +300% (column) from the optimal value. The values for sum of squared difference are normalized by the value corresponding to optimal values for parameters.
(TIF)

Figure S2 **Partial sensitivity analysis on model parameters for parental cell line culture.** Each colormap represents the normalized sum of squared difference between the simulated and measured extracellular metabolite concentration over time, when the parameter (row) is changed from −85% to +300% (column) of the optimal value. The values for sum of squared difference are normalized by the value corresponding to optimal values for parameters. The number for each row corresponds to the parameter presented next to the same row in Figure S1.
(TIF)

Figure S3 **Parameter estimates with their error bars for sensitive parameters.** Glycolysis (A), TCA cycle and Redox state (B), glutaminolysis and pentose phosphate pathway (C), amino acids metabolism (D), energetic (E) and growth (F). Horizontal solid lines are 1.96 standard error bars and represent parameter estimate ±1.96 standard error. Parental cell line: open triangles for parameter estimates, induced low-producer cell line: open squares for parameter estimates, and induced high-producer cell line: open circles for parameter estimates. A parameter is considered highly sensitive if a small variation in its value (±25%) causes more than a 15% increase of in the objective function.
(TIF)

Figure S4 **Comparison of model simulations regarding enzymatic regulation for parental culture for extracellular and energetic metabolites.** Same conditions as in Figure 2 applied.
(TIF)

Figure S5 **Comparison of model simulations regarding enzymatic regulation for parental culture for intracellular metabolites.** Same conditions as in Figure 2 applied.
(TIF)

Figure S6 **Comparison of model simulations regarding enzymatic regulation for induced low-producing culture for extracellular and energetic metabolites.** Same conditions as in Figure 2 applied.
(TIF)

Figure S7 **Comparison of model simulations regarding enzymatic regulation for induced low-producing culture for intracellular metabolites.** Same conditions as in Figure 2 applied.
(TIF)

Figure S8 **Comparison of model simulations regarding enzymatic regulation for induced high-producing culture for extracellular and energetic metabolites.** Same conditions as in Figure 2 applied.
(TIF)

Figure S9 **Comparison of model simulations regarding enzymatic regulation for induced high-producing culture for intracellular metabolites.** Same conditions as in Figure 2 applied.
(TIF)

Figure S10 **Simulated and experimental data for parental and induced/non-induced cell line**. Parental (experimental data: black triangles, simulated data: solid black line), induced low-producer (experimental data: black squares, simulated data: dashed black line), non-induced low producer (experimental data: blue squares, simulated data: dashed blue line), induced high-producer (experimental data: black circles, simulated data: dotted black line), and non-induced high-producer (experimental data: red circles, simulated data: dotted red line).
(TIF)

Table S1 **MRM transition and retention time of each amino acid quantified.**
(DOCX)

Table S2 **MRM mode with the mass spectrometer conditions for determination of nucleotides.**
(DOCX)

Table S3 **MRM mode with the mass spectrometer conditions for determination of nucleotides.**
(DOCX)

Table S4 **Reactions of the metabolic network.**
(DOCX)

Table S5 **Biokinetic equations of the metabolites fluxes (1-35) of the model.**
(DOCX)

Table S6 **State variables description and initial conditions.**
(DOCX)

Table S7 **Affinity (Km), activation (Ka), and inhibition (Ki) constants.**
(DOCX)

Table S8 **Maximum reaction rates (νmax) and comparison of highly sensitive parameters in parental, low-producing and high-producing clones.**
(DOCX)
