# Correction: Analyzing Clonal Variation of Monoclonal Antibody-Producing CHO Cell Lines Using an *In Silico* Metabolomic Platform

**DOI:** 10.1371/journal.pone.0104725

**Published:** 2014-08-01

**Authors:** The PLOS ONE Staff

There is an error in Figure 2. The arrow representing activation term IV is incorrectly directed towards the PGK enzyme; it should be directed towards the PK enzyme. Please see the correct Figure 2 here.

**Figure 2:**
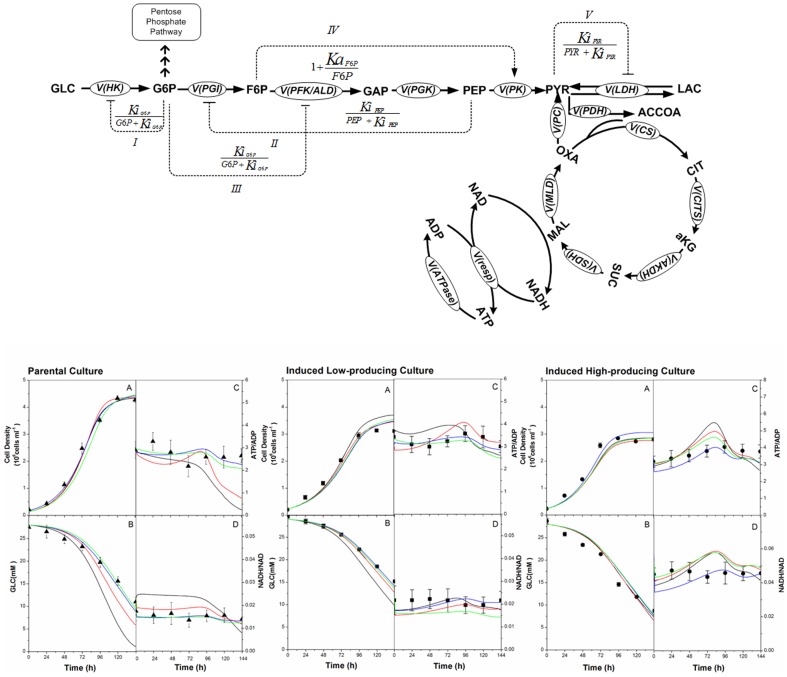
Regulation scheme of the model with enzymes activation or inhibition. Symbol “↓” indicates activation and “⊥” inhibition. Glycolytic enzymes are either inhibited 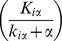
or activated 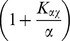
by an effector “α”. The corresponding activation/inhibition terms are labeled as I, II, III, IV, and V. The bottom diagram represents model simulations for parental, induced low- and induced high-producer cell lines with no regulation (solid black line), with the addition of term I (solid red line), with the addition of terms I and II (solid blue line), and the addition of all terms (solid green line). Experimental data are represented by triangles (parental culture), squares (induced low-producing culture), and circles (induced high-producing culture) for cell density (A), glucose (B), ATP-to-ADP ratio (C), and NADH-to-NAD ratio (D). Error bars are standard deviations from duplicate flasks. Error bars are standard deviations for duplicate cultures.

## References

[1] Ghorbaniaghdam A, Chen J, Henry O, Jolicoeur M (2014) Analyzing Clonal Variation of Monoclonal Antibody-Producing CHO Cell Lines Using an *In Silico* Metabolomic Platform. PLoS ONE 9(3): e90832 doi:10.1371/journal.pone.0090832 24632968 10.1371/journal.pone.0090832 PMC3954614

