# Supplementary figures and images for: Analyzing Clonal Variation of Monoclonal Antibody-Producing CHO Cell Lines Using an In Silico Metabolomic Platform
Source: PLoS One. 2014 Mar 14;9(3):e90832. doi: 10.1371/journal.pone.0090832 (PMC3954614; doi:10.1371/journal.pone.0090832)

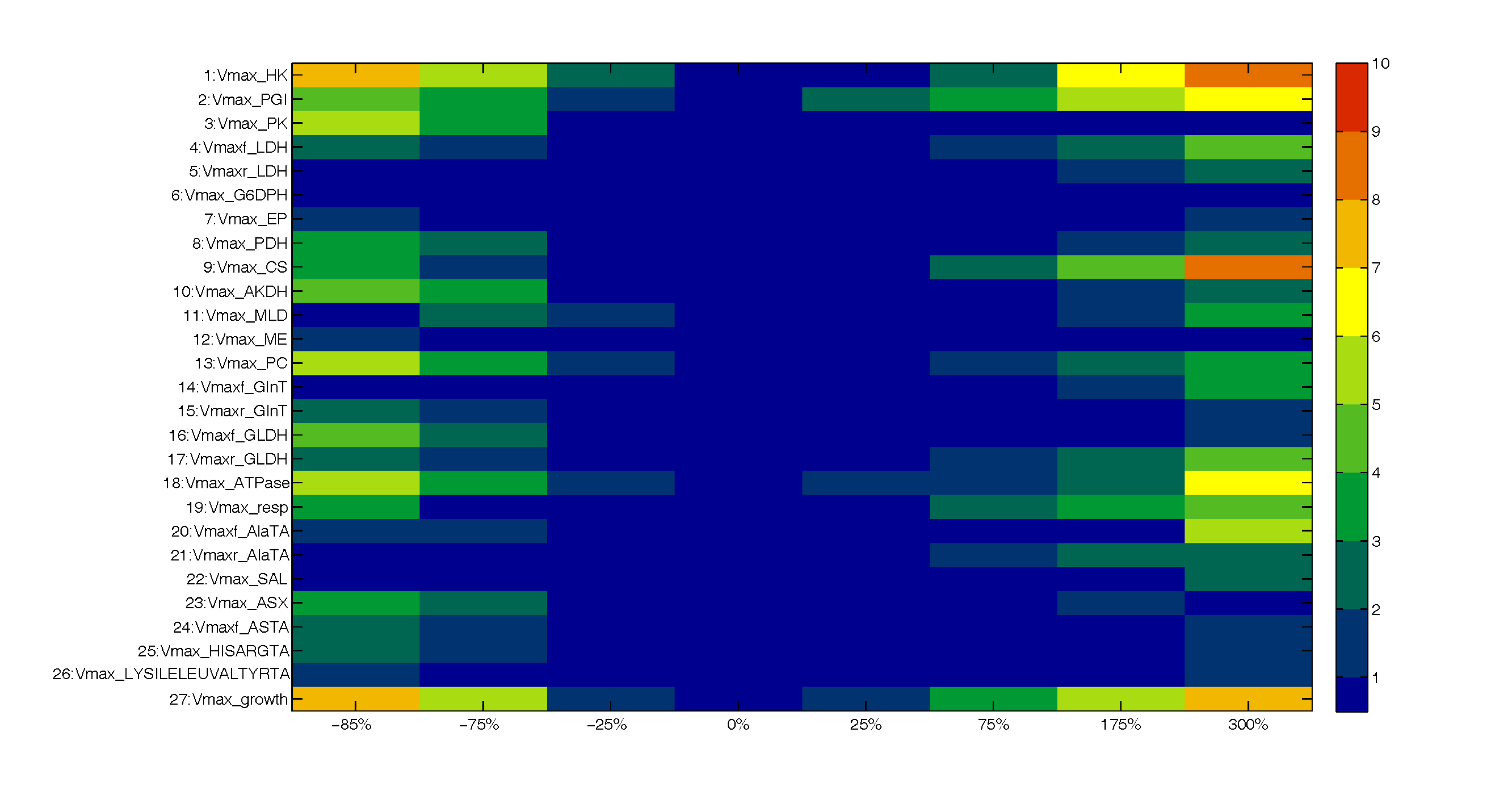

Supplement: Figure S1 — Sensitivity analysis on model parameters for parental cell line culture. The colormap represents the normalized sum of squared difference between model simulations and experimental data, when the parameter (row) is changed from −85% to +300% (column) from the optimal value. The values for sum of squared difference are normalized by the value corresponding to optimal values for parameters. (TIF) [file pone.0090832.s001.tif]

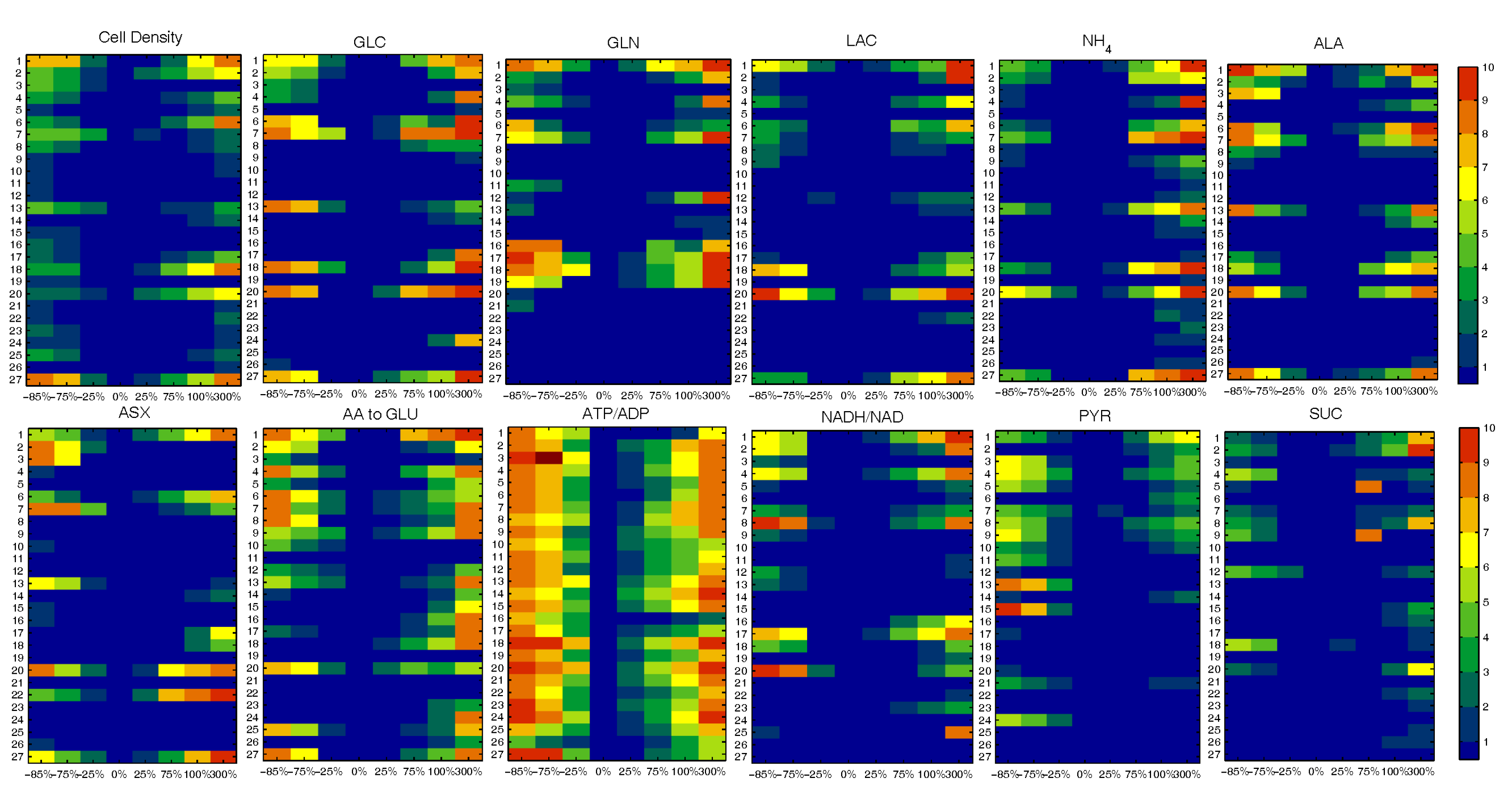

Supplement: Figure S2 — Partial sensitivity analysis on model parameters for parental cell line culture. Each colormap represents the normalized sum of squared difference between the simulated and measured extracellular metabolite concentration over time, when the parameter (row) is changed from −85% to +300% (column) of the optimal value. The values for sum of squared difference are normalized by the value corresponding to optimal values for parameters. The number for each row corresponds to the parameter presented next to the same row in Figure S1. (TIF) [file pone.0090832.s002.tif]

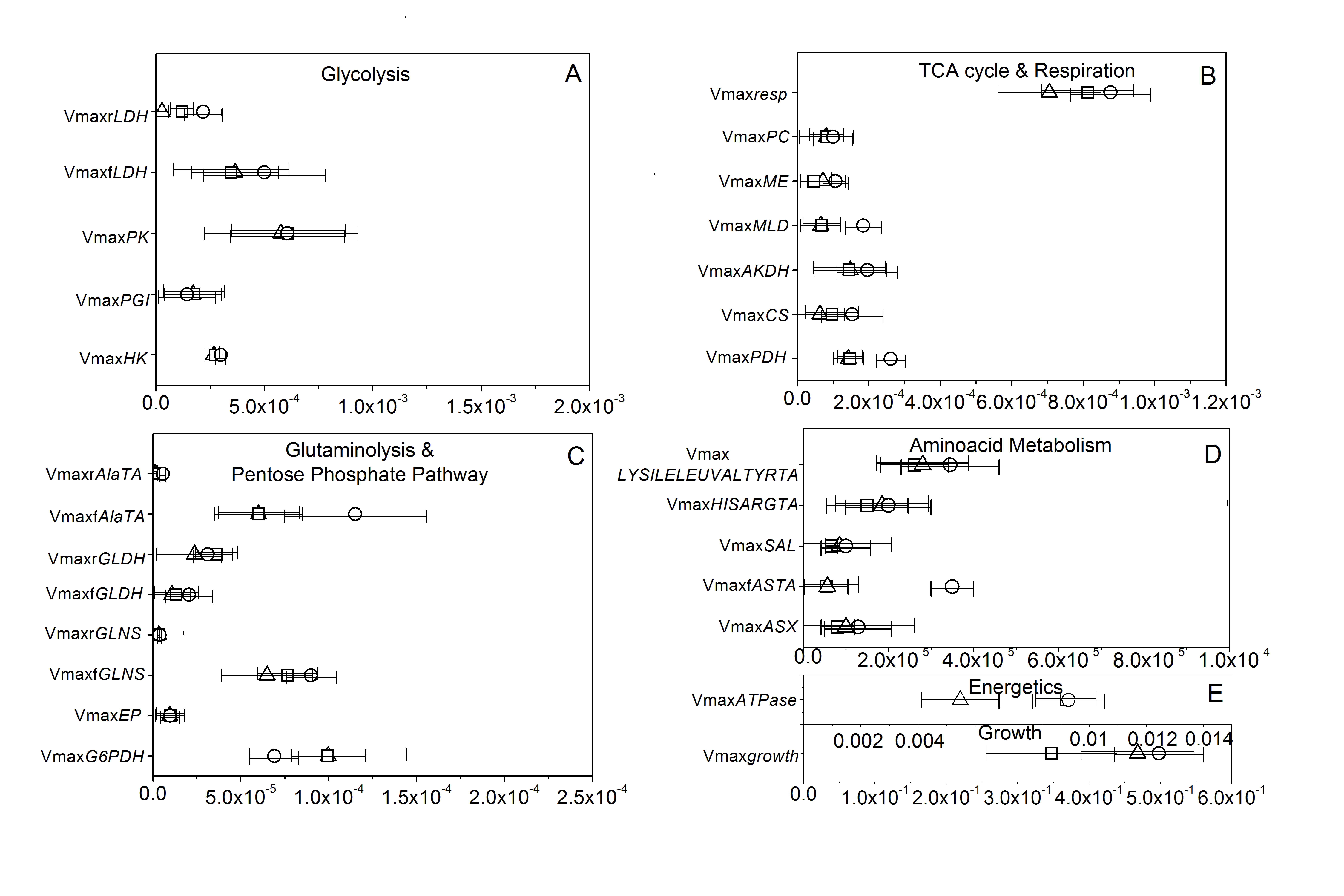

Supplement: Figure S3 — Parameter estimates with their error bars for sensitive parameters. Glycolysis (A), TCA cycle and Redox state (B), glutaminolysis and pentose phosphate pathway (C), amino acids metabolism (D), energetic (E) and growth (F). Horizontal solid lines are 1.96 standard error bars and represent parameter estimate ±1.96 standard error. Parental cell line: open triangles for parameter estimates, induced low-producer cell line: open squares for parameter estimates, and induced high-producer cell line: open circles for parameter estimates. A parameter is considered highly sensitive if a small variation in its value (±25%) causes more than a 15% increase of in the objective function. (TIF) [file pone.0090832.s003.tif]
