# Supplementary material for: Analyzing Clonal Variation of Monoclonal Antibody-Producing CHO Cell Lines Using an In Silico Metabolomic Platform
Source: PLoS One. 2014 Mar 14;9(3):e90832. doi: 10.1371/journal.pone.0090832 (PMC3954614; doi:10.1371/journal.pone.0090832)
Supplement: Table S1 — MRM transition and retention time of each amino acid quantified. (DOCX) [file pone.0090832.s011.docx]

**Table S1.** MRM transition and retention time of each amino acid quantified

| **Compound name** | **IS**  **(**Internal standard) | **Precursor ion**  **(M/Z)** | **Product ion**  **(M/Z)** | **Dwell**  **(ms)** | **Fragment**  **(v)** | **CE**  **(v)** | **RT**  **(min)** |
| --- | --- | --- | --- | --- | --- | --- | --- |
| Cystine | NO | 241.3 | 241.3 | 100 | 76 | 0 | 14.78 |
| homoarginine | Yes | 189.2 | 144 | 100 | 92 | 12 | 14.44 |
| tyrosine | NO | 182.2 | 136.1 | 100 | 66 | 8 | 8.74 |
| homophenylalanine | yes | 180.2 | 134.1 | 100 | 75 | 8 | 6.12 |
| arginine | NO | 175.2 | 70.1 | 100 | 95 | 24 | 15.09 |
| phenylalanine | NO | 166.2 | 120.1 | 100 | 72 | 8 | 6.92 |
| histidine | NO | 156.2 | 110.1 | 100 | 81 | 12 | 16.84 |
| Methionine-d3 | yes | 153.2 | 136.1 | 100 | 69 | 4 | 8.22 |
| methionine | NO | 150.2 | 133 | 100 | 63 | 4 | 8.18 |
| Glutamic acid | NO | 148.1 | 84.1 | 100 | 72 | 16 | 12.59 |
| lysine | NO | 147.2 | 84.1 | 100 | 66 | 12 | 15.48 |
| glutamine | NO | 147.2 | 84.1 | 100 | 72 | 16 | 11.3 |
| Aspartic acid | NO | 134.1 | 74.1 | 100 | 61 | 12 | 14.23 |
| asparagine | NO | 133.1 | 74 | 100 | 60 | 12 | 11.87 |
| isoleucine | NO | 132.2 | 86.1 | 100 | 63 | 4 | 7.431 |
| leucine | NO | 132.2 | 86.1 | 100 | 72 | 4 | 7.431 |
| threonine | NO | 120.1 | 103.1 | 100 | 133 | 16 | 6.94 |
| valine | NO | 118.2 | 72.1 | 100 | 55 | 8 | 9 |
| proline | NO | 116.1 | 70.1 | 100 | 75 | 12 | 9.6 |
| serine | NO | 106.1 | 60.1 | 100 | 60 | 8 | 11.62 |
| alanine | NO | 90.1 | 44.1 | 100 | 42 | 8 | 10.7 |
| glycine | NO | 76.1 | 30.1 | 100 | 39 | 4 | 11.47 |
