# Supplementary material for: Analyzing Clonal Variation of Monoclonal Antibody-Producing CHO Cell Lines Using an In Silico Metabolomic Platform
Source: PLoS One. 2014 Mar 14;9(3):e90832. doi: 10.1371/journal.pone.0090832 (PMC3954614; doi:10.1371/journal.pone.0090832)
Supplement: Table S2 — MRM mode with the mass spectrometer conditions for determination of nucleotides. (DOCX) [file pone.0090832.s012.docx]

**Table S2.** MRM mode with the mass spectrometer conditions for determination of nucleotides

| **Nucleotides** | **Precursor ion**  **(M/Z)** | **Product ion**  **(M/Z)** | **Dwell**  **(ms)** | **Fragment**  **(v)** | **CE**  **(v)** |
| --- | --- | --- | --- | --- | --- |
| NADPH | 746 | 136 | 100 | 192 | 68 |
| UTP | 743 | 743 | 100 | 169 | 0 |
| UDPG | 589 | 426.9 | 100 | 195 | 10 |
| GTP | 524 | 152 | 100 | 215 | 26 |
| ATP | 508 | 136 | 100 | 220 | 38 |
| CTP | 484 | 112 | 100 | 200 | 18 |
| ADP | 428 | 136 | 100 | 177 | 28 |
| AMP | 348 | 136 | 100 | 144 | 14 |
