# Supplementary material for: Analyzing Clonal Variation of Monoclonal Antibody-Producing CHO Cell Lines Using an In Silico Metabolomic Platform
Source: PLoS One. 2014 Mar 14;9(3):e90832. doi: 10.1371/journal.pone.0090832 (PMC3954614; doi:10.1371/journal.pone.0090832)
Supplement: Table S3 — MRM mode with the mass spectrometer conditions for determination of nucleotides. (DOCX) [file pone.0090832.s013.docx]

**Table S3.** MRM mode with the mass spectrometer conditions for determination of nucleotides

| **Compound name** | **Precursor ion**  **(M/Z)** | **Product ion**  **(M/Z)** | **Dwell**  **(ms)** | **Fragment**  **(v)** | **CE**  **(v)** |
| --- | --- | --- | --- | --- | --- |
| F6P | 259 | 96.9 | 100 | 94 | 12 |
| G6P | 259 | 96.9 | 100 | 94 | 8 |
| G1P | 259 | 79 | 100 | 94 | 24 |
| X5P | 229 | 97 | 100 | 78 | 4 |
| R5P | 229 | 78.9 | 100 | 72 | 32 |
| E4P | 199 | 97 | 100 | 73 | 4 |
| PEP | 167 | 79 | 100 | 60 | 12 |
| Alpha_keto | 145 | 101 | 100 | 60 | 4 |
| Malic acid | 133 | 71 | 100 | 72 | 12 |
| Succ acid | 117 | 73 | 100 | 63 | 8 |
| Fumaric acid | 115 | 70.9 | 100 | 63 | 4 |
| pyruvate | 87 | 87 | 100 | 51 | 0 |
