# Supplementary material for: Analyzing Clonal Variation of Monoclonal Antibody-Producing CHO Cell Lines Using an In Silico Metabolomic Platform
Source: PLoS One. 2014 Mar 14;9(3):e90832. doi: 10.1371/journal.pone.0090832 (PMC3954614; doi:10.1371/journal.pone.0090832)
Supplement: Table S4 — Reactions of the metabolic network. (DOCX) [file pone.0090832.s014.docx]

**Table S4. Reactions of the metabolic network**

| No. | Reaction |
| --- | --- |
| 1 GLC+ATP→G6P+ADP | |
| 2 G6P→F6P | |
| 3 | F6P+ATP→2GAP+ADP |
| 4 | GAP+ADP+NAD^+^+Pi→PEP+ATP+NADH |
| 5 | PEP+ADP→PYR+ATP |
| 6 | PYR+NADH↔LAC+NAD^+^ |
| 7 | G6P+2NADP^+^→R5P+2NADPH+CO_2_ |
| 8 | R5P→X5P |
| 9 | R5P+2X5P→2F6P+GAP |
| 10 | PYR+COA+NAD^+^→ACCOA+NADH+CO_2_ |
| 11 | ACCOA+OXA→CIT+CoA |
| 12 | CIT+NAD^+^→AKG+NADH+CO_2_ |
| 13 | AKG+CoA+NAD^+^+ADP+Pi →SUC+NADH+CO_2_+CoA+ATP |
| 14 | SUC+2/3NAD^+^→MAL+2/3NADH+CO_2_ |
| 15 | MAL+NAD^+^→OAA+NADH |
| 16 | MAL+NADP →PYR+NADPH+CO_2_ |
| 17 | PYR →OAA+CO_2_ |
| 18 | GLN+ATP↔GLU+ADP+NH_4_ |
| 19 | GLU+NAD^+^↔AKG+NADH+NH_4_ |
| 20 | GLU+PYR↔AKG+ALA |
| 21 | GLU+ADP+Pi→EGLU+ATP |
| 22 | O_2_+(P/O ratio)*2ADP+2NADH+(P/O ratio)*2Pi→(P/O ratio)*2ATP+NAD^+^+2H_2_O |
| 23 | O_2_+2NADH→2NAD^+^+2H_2_O |
| 24 | ATP→ADP+Pi |
| 25 | ATP+AMP↔2ADP |
| 26 | Pcr+ADP↔Cr+ATP |
| 27 | 2GLN+0.6R5P+2ASP+GLY+2ATP→2GLU+2MAL+AMP+2ADP |
| 28 | NADPH→NADP^+^ |
| 29 | SER→PYR+ NH_4_ |
| 30 | ASX→ASP+ NH_4_ |
| 31 | ASP+AKG↔GLU+OAA+NH_4_ |
| 32 | HIS+ARG+AKG→GLU+NH_4_+CO_2_ |
| 33 | LYS+ILE+LEU+HIS+VAL+TYR+7AKG+ATP+9NAD+2NADP→4GLU+3SUC+MAL+8ACCOA+ADP+9NADH+2NADPH+4CO_2_ |
| 34 | 0.024R5P+0.029G6P+0.04GLN+0.013ALA+0.007ARG+0.0261ASP+0.003HIS+0.0084ILE+0.013LEU+0.01LYS+0.099SER+0.004TYR+0.0096VAL+0.016GLY+3.78ATP→X |
| 35 | 0.01GLU+0.01GLN+0.01ALA+0.005ARG+0.007ASN+0.008ASP+0.003HIS+0.005ILE+0.014LEU+0.014LYS+0.026SER+0.008TYR+0.018VAL+0.0145GLY+4ATP→mAb |
