# Supplementary material for: Analyzing Clonal Variation of Monoclonal Antibody-Producing CHO Cell Lines Using an In Silico Metabolomic Platform
Source: PLoS One. 2014 Mar 14;9(3):e90832. doi: 10.1371/journal.pone.0090832 (PMC3954614; doi:10.1371/journal.pone.0090832)
Supplement: Table S5 — Biokinetic equations of the metabolites fluxes (1-35) of the model. (DOCX) [file pone.0090832.s015.docx]

**Table S5.** **Biokinetic equations of the metabolites fluxes (1-35) of the model**

| No. | Biokinetic equations |
| --- | --- |
| 1 | $\nu\left( HK \right)=\nu_{\max HK}*\frac{GLC}{K_{mGLC}+GLC}*\frac{\frac{ATP}{ADP}}{K_{m\frac{ATP}{ADP}}+\frac{ATP}{ADP}}*\frac{K_{iG6P}}{K_{iG6P}+G6P}$ |
| 2 | $\nu\left( PGI \right)=\nu_{maxf PGI}*\frac{G6P}{K_{mG6P}+G6P}*\frac{K_{iPEP}}{K_{iPEP}+PEP}$ |
| 3 | $\nu\left( PFK/ALD \right)=\nu_{\max PFK/ELD}*\frac{F6P}{K_{mF6P}+F6P}*\frac{\frac{ATP}{ADP}}{K_{m\frac{ATP}{ADP}}+\frac{ATP}{ADP}}*\frac{K_{iG6P}}{K_{iG6P}+G6P}$ |
| 4 | $\nu\left( PGK \right)=\nu_{maxPGK}*\frac{GAP}{K_{mGAP}+GAP}*\frac{\frac{ADP}{ATP}}{K_{m\frac{ADP}{ATP}}+\frac{ADP}{ATP}}*\frac{Pi}{K_{mPi}+Pi}$*$\frac{\frac{NAD}{NADH}}{K_{m\frac{NAD}{NADH}}+ \frac{NAD}{NADH}}$ |
| 5 | $\nu\left( PK \right)=\nu_{\max PK}*\frac{PEP}{K_{mPEP}*(1+\frac{{Ka}_{F6P}}{F6P})+PEP}*\frac{\frac{ADP}{ATP}}{K_{m\frac{ADP}{ATP}}+\frac{ADP}{ATP}}$ |
| 6 | $\nu\left( LDH \right)=\nu_{maxf LDH}*\frac{PYR}{K_{mPYR}+PYR}*\frac{\frac{NADH}{NAD}}{K_{m\frac{NADH}{NAD}}+\frac{NADH}{NAD}}-\nu_{maxr LDH}*\frac{{LAC}^{.}}{K_{mLAC}+LAC}*\frac{\frac{NAD}{NADH}}{K_{m\frac{NAD}{NADH}}+\frac{NAD}{NADH}}*\frac{K_{iPYR}}{K_{iPYR}+PYR}$ |
| 7 | $\nu\left( G6PDH/PGLcDH \right)=\nu_{\max G6PDH/PGLcDH}*\frac{G6P}{K_{mG6P}+G6P}*\frac{\frac{NADP}{NADPH}}{K_{m\frac{NADP}{NADPH}}+\frac{NADP}{NADPH}}$ |
| 8 | $\nu\left( EP \right)=\nu_{\max EP}*\frac{R5P}{K_{mR5P}+R5P}$ |
| 9 | $\nu\left( TK/TA \right)=\nu_{\max TK/TA}*\frac{R5P}{K_{m2R5P}+R5P}*\frac{X5P}{K_{mX5P}+X5P}$ |
| 10 | $\nu\left( PDH \right)=\nu_{max PDH}*\frac{PYR}{K_{mPYR}+PYR}*\frac{\frac{NAD}{NADH}}{K_{m\frac{NAD}{NADH}}+\frac{NAD}{NADH}}$ |
| 11 | $\nu\left( CS \right)=\nu_{max CS}*\frac{ACCOA}{K_{mACCOA}+ACCOA}*\frac{OAA}{K_{mOXA}+OAA}$ |
| 12 | $\nu\left( CITS/ISOD \right)=\nu_{\max CITS/ISOD}*\frac{CIT}{K_{mCIT}+CIT}*\frac{\frac{NAD}{NADH}}{K_{m\frac{NAD}{NADH}}+\frac{NAD}{NADH}}$ |

**Table S5.** **Biokinetic equations of the metabolites fluxes (1-35) of the model (continued)**

| 13 | $\boldsymbol{\nu}\left( \boldsymbol{AKGDH} \right)\boldsymbol{=}\boldsymbol{\nu}_{\boldsymbol{max AKGDH}}\boldsymbol{*}\frac{\boldsymbol{AKG}}{\boldsymbol{K}_{\boldsymbol{mAKG}}\boldsymbol{+AKG}}\boldsymbol{*}\frac{\frac{\boldsymbol{NAD}}{\boldsymbol{NADH}}}{\boldsymbol{K}_{\boldsymbol{m}\frac{\boldsymbol{NAD}}{\boldsymbol{NADH}}}\boldsymbol{+}\frac{\boldsymbol{NAD}}{\boldsymbol{NADH}}}\boldsymbol{*}\frac{\frac{\boldsymbol{ADP}}{\boldsymbol{ATP}}}{\boldsymbol{K}_{\boldsymbol{m}\frac{\boldsymbol{ADP}}{\boldsymbol{ATP}}}\boldsymbol{+}\frac{\boldsymbol{ADP}}{\boldsymbol{ATP}}}\boldsymbol{*}\frac{\boldsymbol{Pi}}{\boldsymbol{K}_{\boldsymbol{mPi}}\boldsymbol{+Pi}}$ |
| --- | --- |
| 14 | $\nu\left( SDH/FUM \right)=\nu_{\max SDH/FUM}*\frac{SUC}{K_{mSUC}+SUC}*\frac{\frac{NAD}{NADH}}{K_{m\frac{NAD}{NADH}}+\frac{NAD}{NADH}}$ |
| 15 | $\nu\left( MDH \right)=\nu_{max MDH}*\frac{MAL}{K_{mMAL}+MAL}*\frac{\frac{NAD}{NADH}}{K_{m\frac{NAD}{NADH}}+\frac{NAD}{NADH}}$ |
| 16 | $\nu(ME)=\nu_{max ME}*\frac{MAL}{K_{mMAL}+MAL}*\frac{\frac{NAPD}{NADPH}}{K_{m\frac{NADP}{NADPH}}+\frac{NADP}{NADPH}}$ |
| 17 | $\nu(PC)=\nu_{max ME}*\frac{PYR}{K_{mPYR}+PYR}$ |
| 18 | $\nu\left( GLNS \right)=\nu_{maxfGLNS}*\frac{GLN}{K_{mGLN}+GLN}*\frac{\frac{ATP}{ADP}}{K_{m\frac{ATP}{ADP}}+\frac{ATP}{ADP}}-\nu_{maxr GLNS}*\frac{GLU}{K_{mGLU}+GLU}*\frac{\frac{ADP}{ATP}}{K_{m\frac{ADP}{ATP}}+\frac{ADP}{ATP}}*\frac{{NH}_{4}}{K_{m{NH}_{4}}+{NH}_{4}}$ |
| 19 | $\nu\left( GLDH \right)=\nu_{maxf GLDH}*\frac{GLU}{K_{mGLU}+GLU}*\frac{\frac{NAD}{NADH}}{K_{m\frac{NAD}{NADH}}+\frac{NAD}{NADH}}-\nu_{maxr GLDH}*\frac{AKG}{K_{mAKG}+AKG}*\frac{\frac{NADH}{NAD}}{K_{m\frac{NADH}{NAD}}+\frac{NADH}{NAD}}*\frac{{NH}_{4}}{K_{m{NH}_{4}}+{NH}_{4}}$ |
| 20 | $\nu\left( AlaTA \right)=\nu_{maxf AlaTA}*\frac{GLU}{K_{mGLU}+GLU}*\frac{PYR}{K_{mPYR}+PYR}-\nu_{maxr AlaTA}*\frac{ALA}{K_{mALA}+ALA}*\frac{AKG}{K_{mPYR}+AKG}*(1+\frac{K_{aGLN}}{GLN})$ |
| 21 | $\nu\left( GluT \right)=\nu_{maxGluT}*\frac{GLU}{K_{mGLU}+GLU}*\frac{\frac{ADP}{ATP}}{K_{m\frac{ADP}{ATP}}+\frac{ADP}{ATP}}$*$\frac{Pi}{K_{mPi}+Pi}$ |
| 22 | $\nu\left( resp \right)=\nu_{max resp}*\frac{O_{2}}{K_{mO_{2}}+O_{2}}*\frac{\frac{ADP}{ATP}}{K_{m\frac{ADP}{ATP}}+\frac{ADP}{ATP}}*\frac{NADH}{K_{mNADH}+NADH}*\frac{Pi}{K_{mPi}+Pi}$ |
| 23 | $\nu\left( leak \right)=\nu_{max leak}\frac{NADH}{K_{mNADH}+NADH}$ |
| 24 | $\nu\left( ATPase \right)=\nu_{max ATPase}*\frac{ATP}{ATP+ATP}$ |

**Table S5.** **Biokinetic equations of the metabolites fluxes (1-35) of the model (continued)**

| 25 | $\boldsymbol{\nu}\left( \boldsymbol{AK} \right)\boldsymbol{=}\boldsymbol{\nu}_{\boldsymbol{maxf AK}}\boldsymbol{*}\frac{\boldsymbol{ATP}}{\boldsymbol{K}_{\boldsymbol{mATP}}\boldsymbol{+ATP}}\boldsymbol{*}\frac{\boldsymbol{AMP}}{\boldsymbol{K}_{\boldsymbol{mAMP}}\boldsymbol{+AMP}}\boldsymbol{-}\boldsymbol{\nu}_{\boldsymbol{maxr AK}}\boldsymbol{*}\frac{\boldsymbol{ADP}}{\boldsymbol{K}_{\boldsymbol{mADP}}\boldsymbol{+ADP}}$ |
| --- | --- |
| 26 | $\nu\left( CK \right)=\nu_{maxf CK}*\frac{ADP}{K_{mADP}+ADP}*\frac{Pcr}{K_{mPcr}+Pcr}-\nu_{maxr CK}*\frac{ATP}{K_{mATP}+ATP}*\frac{Cr}{K_{mCr}+Cr}$ |
| 27 | $\nu\left( PPRiBP \right)=\nu_{\max PPRibP}*\frac{R5P}{K_{mR5P}+R5P}*\frac{ASP}{K_{mASP}+ASP}*\frac{GLN}{K_{mGLN}+GLN}*\frac{GLY}{K_{mGLY}+GLY}$ |
| 28 | $\nu\left( NADPHox \right)=\nu_{\max NADPHox}*\frac{NADPH}{K_{mNADPH}+NADPH}$ |
| 29 | $\nu\left( SAL \right)=\nu_{max SAL}*\frac{SER}{K_{mSER}+SER}$ |
| 30 | $\nu\left( ASX \right)=\nu_{max ASX}*\frac{ASX}{K_{mASX}+ASX}$ |
| 31 | $\nu\left( ASTA \right)=\nu_{maxf ASTA}*\frac{ASP}{K_{mASP}+ASP}*\frac{AKG}{K_{mAKG}+AKG}-\nu_{maxr ASTA}*\frac{GLU}{K_{mGLU}+GLU}*\frac{OAA}{K_{mOXA}+OAA}*\frac{{NH}_{4}}{K_{m{NH}_{4}}+{NH}_{4}}$ |
| 32 | $\nu\left( HISARGTA \right)=\nu_{\max HISARGTA}*\frac{HIS}{K_{mHIS}+HIS}*\frac{ARG}{K_{mARG}+ARG}*\frac{AKG}{K_{mAKG}+AKG}$ |
| 33 | $\nu\left( LYSILELEUHISVALTYRTA \right)=\nu_{\max groLYSILELEUVALTYRTAwth}*\frac{LYS}{K_{mLYS}+LYS}*\frac{ILE}{K_{mILE}+ILE}*\frac{LEU}{K_{mLEU}+LEU}$  $*\frac{VAL}{K_{mVAL}+VAL}*\frac{HIS}{K_{mHIS}+HIS}*\frac{TYR}{K_{mTYR}+TYR}**\frac{AKG}{K_{mAKG}+AKG}*\frac{\frac{ATP}{ADP}}{K_{m\frac{ATP}{ADP}}+\frac{ATP}{ADP}}*\frac{\frac{NAD}{NADH}}{K_{m\frac{NAD}{NADH}}+\frac{NAD}{NADH}}*\frac{\frac{NADP}{NADPH}}{K_{m\frac{NADP}{NADPH}}+\frac{NADP}{NADPH}}$ |
| 34 | $\nu\left( growth \right)=\nu_{\max growth}*\frac{R5P}{K_{mR5P}+R5P}*\frac{G6P}{K_{mG6P}+G6P}*\frac{GLN}{K_{mGLN}+GLN}*\frac{ALA}{K_{mALA}+ALA}*\frac{ARG}{K_{mARG}+ARG}**\frac{ASP}{K_{mASP}+ASP}*\frac{HIS}{K_{mHIS}+HIS}*\frac{ILE}{K_{mILE}+ILE}*\frac{LEU}{K_{mLEU}+LEU}*\frac{LYS}{K_{mLYS}+LYS}*\frac{SER}{K_{mSER}+SER}*\frac{TYR}{K_{mTYR}+TYR}*\frac{VAL}{K_{mVAL}+VAL}*\frac{GLY}{K_{mGLY}+GLY}\frac{\frac{ATP}{ADP}}{K_{m\frac{ATP}{ADP}}+\frac{ATP}{ADP}}$ |
| 35 | $\nu\left( mAb \right)=\nu_{maxmAb}*\frac{R5P}{K_{mR5P}+R5P}*\frac{G6P}{K_{mG6P}+G6P}*\frac{GLN}{K_{mGLNmab}+GLN}*\frac{ALA}{K_{mALA}+ALA}*\frac{ARG}{K_{mARG}+ARG}*\frac{ASX}{K_{mASX}+ASX}*\frac{ASP}{K_{mASP}+ASP}*\frac{HIS}{K_{mHIS}+HIS}*\frac{ILE}{K_{mILE}+ILE}*\frac{LEU}{K_{mLEU}+LEU}*\frac{LYS}{K_{mLYS}+LYS}*\frac{SER}{K_{mSER}+SER}*\frac{TYR}{K_{mTYR}+TYR}*\frac{VAL}{K_{mVAL}+VAL}*\frac{GLY}{K_{mGLY}+GLY}\frac{\frac{ATP}{ADP}}{K_{m\frac{ATP}{ADP}}+\frac{ATP}{ADP}}$ |
