# Supplementary material for: Analyzing Clonal Variation of Monoclonal Antibody-Producing CHO Cell Lines Using an In Silico Metabolomic Platform
Source: PLoS One. 2014 Mar 14;9(3):e90832. doi: 10.1371/journal.pone.0090832 (PMC3954614; doi:10.1371/journal.pone.0090832)
Supplement: Table S6 — State variables description and initial conditions. (DOCX) [file pone.0090832.s016.docx]

**Table S6. State variables description and initial conditions**

| Component | Description | Value | Units | Measured |
| --- | --- | --- | --- | --- |
| ACCoA | Acetyl-CoezymeA | 1.4E-7 | mmol.(10^6^ cells)^-1^ | No^3^ |
| AKG | α-Ketoglutarate | 4E-7 | ‘‘ | Yes^2^ |
| ADP | Adenosine Diphosphate | 1.4E-7 | ‘‘ | Yes^1^ |
| AMP | Adenosine Monophosphate | 2E-8 | ‘‘ | Yes^1^ |
| ATP | Adenosine Triphosphate | 1.5E-6 | ‘‘ | Yes^1^ |
| CIT | Citrate | 2E-7 | ‘‘ | No^2^ |
| CoA | CoenzymeA | 2E-8 | ‘‘ | No |
| Cr | Creatine | 4E-6 | ‘‘ | No |
| F6P | Fructose 6-Phosphate | 3E-7 | ‘‘ | No |
| G6P | Glucose 6-Phosphate | 5E-8 | ‘‘ | Yes^2^ |
| GAP | Glyceraldehyde 3-Phosphate | 4E-7 | ‘‘ |  |
| GLU | Glutamate | 7E-4 | ‘‘ | No^3^ |
| GLY | Glycine | 8E-6 | ‘‘ | Yes^2^ |
| MAL | Malate | 1 E-6 | ‘‘ | Yes^1^ |
| NAD | Nicotinamide Adenine Dinucleotide | 9E-7 | ‘‘ | Yes^1^ |
| NADH | Nicotinamide adenine dinucleotide(reduced) | 2E-8 | ‘‘ | Yes^1^ |
| NADP | Nicotinamide Adenine Dinucleotide Phosphate | 9E-13 | ‘‘ | Yes^1^ |
| NADPH | Nicotinamide Adenine Dinucleotide Phosphate(reduced) | 5.3E-12 | ‘‘ | Yes^1^ |
| OXA | Oxaloacetate | 5E-5 | ‘‘ | No^3^ |
| O_2_ | IntracellularOxygen | 8E-6 | ‘‘ | No^3^ |
| PEP | Phosphoenolpyruvate | 4E-8 | ‘‘ | Yes^2^ |
| PCr | PhosphoCreatine | 2E-6 | ‘‘ | No^3^ |
| Pi | Phosphate | 2E-6 | ‘‘ | No^3^ |
| PYR | Pyruvate | 5E-7 | ‘‘ | Yes^2^ |
| R5P | Ribose 5-Phosphate | 1.5E-7 | ‘‘ | Yes^2^ |
| SUC | Succinate | 5.3E-7 | ‘‘ | Yes^2^ |
| X5P | Xylose*-5-*Phosphate | 9.2E-8 | ‘‘ | No |
| ALA | Extracellular Alanine | 0.75 | mM | Yes^2^ |
| ARG | Extracellular Arginine | 2.8 | ‘‘ | Yes^2^ |
| ASP | ExtracellularAspartate | 1 | ‘‘ | Yes^2^ |
| ASX | ExtracellularAspartate and Asparagine | 2.8 | ‘‘ | Yes^2^ |
| GLY | Extracellular Glycine | 1 | ‘‘ | Yes^2^ |
| HIS | Extracellular Histidine | 0.95 | ‘‘ | Yes^2^ |
| ILE | ExtracellularIsoLeucine | 1 | ‘‘ | Yes^2^ |
| LUE | Extracellular Leucine | 0.8 | ‘‘ | Yes^2^ |
| LYS | Extracellular Lysine | 1.2 | ‘‘ | Yes^2^ |
| SER | Extracellular Serine | 2.1 | ‘‘ | Yes^2^ |
| TYR | Extracellular Tyrosine | 0.55 | ‘‘ | Yes^2^ |
| VAL | Extracellular Valine | 0.8 | ‘‘ | Yes^2^ |
| GLC | Extracellular Glucose | 28 | ‘‘ | Yes^1^ |
| GLN | Extracellular Glutamine | 3.6 | ‘‘ | Yes^1^ |
| EGLU | Extracellular Glutamate | 0.65 | ‘‘ | Yes^1^ |
| LAC | Extracellular Lactate | 0.75 | ‘‘ | Yes^1^ |
| NH_4_ | Extracellular Ammonia | 0.5 | ‘‘ | Yes^1^ |
| mAb | Extracellular Monoclonal Antibody | 0 | ‘‘ | Yes |
| X | Cell Density | 0.18E-6 | cells mL^-1^ | Yes |

1 Analytical method: enzyme-based

2 Analytical method: Chemical-based viaUPLC system

3 Database *brenda-enzymes.info* and references therein [48]
