# Supplementary material for: Analyzing Clonal Variation of Monoclonal Antibody-Producing CHO Cell Lines Using an In Silico Metabolomic Platform
Source: PLoS One. 2014 Mar 14;9(3):e90832. doi: 10.1371/journal.pone.0090832 (PMC3954614; doi:10.1371/journal.pone.0090832)
Supplement: Table S7 — Affinity (Km), activation (Ka), and inhibition (Ki) constants. (DOCX) [file pone.0090832.s017.docx]

**Table S7.** Affinity (K_m_), activation (K_a_), and inhibition (K_i_) constants

| Parameter | Value | | | Units |
| --- | --- | --- | --- | --- |
|  | **Parental** | **Low-producing** | **High-producing** |  |
| $\boldsymbol{K}_{\boldsymbol{mGLC}}$ | 4.19 | 4.19 | 4.199 | mM |
| $\boldsymbol{K}_{\boldsymbol{mGLN}}$ | 1.58 | 1.5893 | 1.5893 | ‘‘ |
| $\boldsymbol{K}_{\boldsymbol{mLAC}}$ | 5 | 5 | 3.5 | ‘‘ |
| $\boldsymbol{K}_{\boldsymbol{m}\boldsymbol{NH}_{\boldsymbol{4}}}$ | 0.5 | 0.5 | 0.5 | ‘‘ |
| $\boldsymbol{K}_{\boldsymbol{mALA}}$ | 1 | 1 | 1 | ‘‘ |
| $\boldsymbol{K}_{\boldsymbol{mARG}}$ | 0.05 | 0.05 | 0.05 | ‘‘ |
| $\boldsymbol{K}_{\boldsymbol{mASP}}$ | 0.015 | 0.015 | 0.015 | ‘‘ |
| $\boldsymbol{K}_{\boldsymbol{mASX}}$ | 0.15 | 0.15 | 0.15 | ‘‘ |
| $\boldsymbol{K}_{\boldsymbol{mGLY}}$ | 0.05 | 0.05 | 0.05 | ‘‘ |
| $\boldsymbol{K}_{\boldsymbol{mHIS}}$ | 0.5 | 0.5 | 0.5 | ‘‘ |
| $\boldsymbol{K}_{\boldsymbol{mILE}}$ | 0.025 | 0.025 | 0.025 | ‘‘ |
| $\boldsymbol{K}_{\boldsymbol{mLUE}}$ | 0.025 | 0.025 | 0.025 | ‘‘ |
| $\boldsymbol{K}_{\boldsymbol{mLYS}}$ | 0.05 | 0.05 | 0.05 | ‘‘ |
| $\boldsymbol{K}_{\boldsymbol{mSER}}$ | 0.015 | 0.015 | 0.015 | ‘‘ |
| $\boldsymbol{K}_{\boldsymbol{mTYR}}$ | 0.05 | 0.05 | 0.05 | ‘‘ |
| $\boldsymbol{K}_{\boldsymbol{mVAL}}$ | 0.05 | 0.05 | 0.05 | ‘‘ |
| $\boldsymbol{K}_{\boldsymbol{aGLN}}$ | 10 | 10 | 10 | ‘‘ |
| $\boldsymbol{K}_{\boldsymbol{mGLNmAb}}$ | 0.01 | 0.01 | 0.01 |  |
| $\boldsymbol{K}_{\boldsymbol{mG}\boldsymbol{6}\boldsymbol{P}}$ | 8.20E-08 | 8.20E-08 | 8.20E-08 | mmol.(10^6^ cells)^-1^ |
| $\boldsymbol{K}_{\boldsymbol{mF}\boldsymbol{6}\boldsymbol{P}}$ | 4.00E-07 | 4.00E-07 | 3.22E-07 | ‘‘ |
| $\boldsymbol{K}_{\boldsymbol{mGAP}}$ | 8.00E-07 | 8.00E-07 | 8.00E-07 | ‘‘ |
| $\boldsymbol{K}_{\boldsymbol{mPEP}}$ | 1.70E-07 | 1.73E-07 | 1.70E-07 | ‘‘ |
| $\boldsymbol{K}_{\boldsymbol{mR}\boldsymbol{5}\boldsymbol{P}}$ | 1.30E-08 | 1.35E-08 | 1.35E-08 | ‘‘ |
| $\boldsymbol{K}_{\boldsymbol{mX}\boldsymbol{5}\boldsymbol{P}}$ | 3.40E-08 | 3.40E-08 | 3.40E-08 | ‘‘ |
| $\boldsymbol{K}_{\boldsymbol{mPYR}}$ | 9.80E-07 | 7.98E-07 | 9.80E-08 | ‘‘ |
| $\boldsymbol{K}_{\boldsymbol{mACCOA}}$ | 9.60E-07 | 9.60E-07 | 9.80E-07 | ‘‘ |
| $\boldsymbol{K}_{\boldsymbol{mOXA}}$ | 2.40E-07 | 2.40E-07 | 2.46E-07 | ‘‘ |
| $\boldsymbol{K}_{\boldsymbol{mCIT}}$ | 1.00E-07 | 8.51E-08 | 9.00E-08 | ‘‘ |
| $\boldsymbol{K}_{\boldsymbol{mAKG}}$ | 8.60E-07 | 9.40E-07 | 7.11E-07 | ‘‘ |
| $\boldsymbol{K}_{\boldsymbol{mSUC}}$ | 2.80E-08 | 2.03E-07 | 1.62E-07 | ‘‘ |
| $\boldsymbol{K}_{\boldsymbol{mMAL}}$ | 8.50E-07 | 8.54E-07 | 6.21E-07 | ‘‘ |
| $\boldsymbol{K}_{\boldsymbol{mGLU}}$ | 1.28E-04 | 1.28E-04 | 1.28E-04 | ‘‘ |
| $\boldsymbol{K}_{\boldsymbol{mATP}}$ | 4.20E-06 | 5.00E-06 | 4.61E-06 | ‘‘ |
| $\boldsymbol{K}_{\boldsymbol{mADP}}$ | 3.65E-07 | 3.65E-07 | 3.65E-07 | ‘‘ |
| $\boldsymbol{K}_{\boldsymbol{mAMP}}$ | 2.52E-08 | 2.52E-08 | 2.52E-08 | ‘‘ |
| $\boldsymbol{K}_{\boldsymbol{mPcr}}$ | 7.70E-6 | 7.70E-06 | 7.70E-06 | ‘‘ |
| $\boldsymbol{K}_{\boldsymbol{mCr}}$ | 6.00E-7 | 6.00E-07 | 6.00E-07 | ‘‘ |
| $\boldsymbol{K}_{\boldsymbol{m}\boldsymbol{O}_{\boldsymbol{2}}}$ | 4.00E-6 | 4.00E-6 | 4.00E-6 | ‘‘ |
| $\boldsymbol{K}_{\boldsymbol{mPi}}$ | 1.00E-6 | 1.00E-6 | 1.00E-6 | ‘‘ |
| $\boldsymbol{K}_{\boldsymbol{mNADH}}$ | 1E-7 | 1E-7 | 1E-7 | ‘‘ |
| $\boldsymbol{K}_{\boldsymbol{mNADPH}}$ | 1E-9 | 1E-9 | 1E-9 |  |
| $\boldsymbol{K}_{\boldsymbol{m}\frac{\boldsymbol{ATP}}{\boldsymbol{ADP}}}$ | 1 | 1 | 1 | unitless |
| $\boldsymbol{K}_{\boldsymbol{m}\frac{\boldsymbol{ADP}}{\boldsymbol{ATP}}}$ | 9E-6 | 9E-6 | 9E-6 | ‘‘ |
| $\boldsymbol{K}_{\boldsymbol{m}\frac{\boldsymbol{NADH}}{\boldsymbol{NAD}}}$ | 0.0035 | 0.0035 | 0.0035 | ‘‘ |
| $\boldsymbol{K}_{\boldsymbol{m}\frac{\boldsymbol{NAD}}{\boldsymbol{NADH}}}$ | 0.5 | 0.5 | 0.5 | ‘‘ |
| $\boldsymbol{K}_{\boldsymbol{m}\frac{\boldsymbol{NADP}}{\boldsymbol{NADPH}}}$ | 1.00E-3 | 1.00E-3 | 1.00E-3 | ‘‘ |
| $\boldsymbol{K}_{\boldsymbol{iG}\boldsymbol{6}\boldsymbol{P}}$ | 5.12E-08 | 5.12E-08 | 5.12E-08 | ‘‘ |
| $\boldsymbol{K}_{\boldsymbol{iPEP}}$ | 2.00E-07 | 2.00E-07 | 2.00E-07 | ‘‘ |
| $\boldsymbol{K}_{\boldsymbol{iPYR}}$ | 3E-08 | 3E-08 | 3E-08 | ‘‘ |
| $\boldsymbol{K}_{\boldsymbol{aF}\boldsymbol{6}\boldsymbol{P}}$ | 1E-06 | 1E-06 | 1E-06 | ‘‘ |
