# Supplementary material for: Analyzing Clonal Variation of Monoclonal Antibody-Producing CHO Cell Lines Using an In Silico Metabolomic Platform
Source: PLoS One. 2014 Mar 14;9(3):e90832. doi: 10.1371/journal.pone.0090832 (PMC3954614; doi:10.1371/journal.pone.0090832)
Supplement: Table S8 — Maximum reaction rates (νmax) and comparison of highly sensitive parameters in parental, low-producing and high-producing clones. (DOCX) [file pone.0090832.s018.docx]

**Table S8** Maximum reaction rates (ν_max_) and comparison of highly sensitive parameters in parental, low-producing and high-producing clones.

| Value | | Parental | Low-producing | High-producing | | t test |
| --- | --- | --- | --- | --- | --- | --- |
| $\boldsymbol{\nu}_{\boldsymbol{maxHK}}$ | | 1.87E-4[1.35E-4,2.35E-4] | 2.73E-4[2.14E-4,3.34E-4] | | 3.32E-4[2.61E-4,3.96E-4] | ⎯ |
| $\boldsymbol{\nu}_{\boldsymbol{max PGI}}$ | | 1.72E-4[3.75E-5,3.05E-4] | 1.76E-4[3.42E-5,3.14E-4] | | 1.47E-4[1.08E-5,2.76E-4] | ⎯ |
| $\boldsymbol{\nu}_{\boldsymbol{max} \boldsymbol{PFK}\boldsymbol{/}\boldsymbol{ALD}}$ | | 2.40E-3 | 2.40E-3 | | 2.40E-3 | ⎯ |
| $\boldsymbol{\nu}_{\boldsymbol{max} \boldsymbol{PGK}}$ | | 5.00E-4 | 5.00E-4 | | 5.00E-4 | ⎯ |
| $\boldsymbol{\nu}_{\boldsymbol{max} \boldsymbol{PK}}$ | | 5.78E-4[3.45E4,6.55E-4] | 6.00E-4[3.48E-4,8.75E-4] | | 5.00E-4[2.44E-4,7.67E-4] | ⎯ |
| $\boldsymbol{\nu}_{\boldsymbol{maxf LDH}}$ | | 3.52E-4[1.5E-4,5.5E-4] | 3.50E-4 [8.15E-5,6.2E-4] | | 5E-4[2.13E-4,7.87E-4] | ⎯ |
| $\boldsymbol{\nu}_{\boldsymbol{maxr LDH}}$ | | 2.95E-5[1.12E-5,4.78E-5] | 1.20E-4[6.78E-5,1.73E-4] | | 1.30E-4[7.91E-5,1.94E-4] | p<0.1 * ^+^ |
| $\boldsymbol{\nu}_{\max\boldsymbol{G}\boldsymbol{6}\boldsymbol{PDH}\boldsymbol{/}\boldsymbol{PGLcDH}}$ | | 1.00E-4 [7.85E-5,1.2E-4] | 9.93E-5[5.46E-5,1.46E-4] | | 6.80E-5[5.48E-5,9.5E-5] | ⎯ |
| $\boldsymbol{\nu}_{\boldsymbol{max} \boldsymbol{EP}}$ | | 9.50E-6[1.53E-6,1.75E-5] | 9.91E-6[1.63E-6,1.82E-5] | | 9.61E-6[3.9E-6,1.54E-5] | ⎯ |
| $\boldsymbol{\nu}_{\max\boldsymbol{TK/TA}}$ | | 2.00E-5 | 2.00E-5 | | 2.00E-5 | ⎯ |
| $\boldsymbol{\nu}_{\boldsymbol{max PDH}}$ | | 1.26E-4[0.84E-5,1.67E-4] | 1.47E-4[1.25E-4,1.61E-4] | | 2.60E-4[2.21E-4,3.04E-4] | p<0.1 ^+^ |
| $\boldsymbol{\nu}_{\boldsymbol{max CS}}$ | | 6.30E-5[1.33E-5,1.13E-4] | 9.67E-5[2.19E-5,1.7E-4] | | 1.52E-4[6.57E-5,2.39E-4] | ⎯ |
| $\boldsymbol{\nu}_{\boldsymbol{max AKGDH}}$ | | 1.50E-4[4.57E-5,2.51E-4] | 1.44E-4[4.34E-5,2.4E-4] | | 1.95E-4 [1.11E-4,2.8E-4] | ⎯ |
| $\boldsymbol{\nu}_{\max\boldsymbol{SDH}\boldsymbol{/}\boldsymbol{FUM}}$ | | 1.950E-4 | 1.950E-4 | | 1.950E-4 | ⎯ |
| $\boldsymbol{\nu}_{\boldsymbol{max} \boldsymbol{MLD}}$ | | 6.01E-5[4.76E-6,1.16E-4] | 6.61E-5[8.45E-6,1.31E-4] | | 1.41E-4[9.13E-5,1.92E-4] | ⎯ |
| $\boldsymbol{\nu}_{\boldsymbol{max ME}}$ | | 6.14E-5[1.82E-5,1.04E-4] | 4.56E-5[1.55E-5,7.58E-4] | | 1.05E-5[3.97E-7,2.88E-5] | ⎯ |
| $\boldsymbol{\nu}_{\boldsymbol{max PC}}$ | | 8.93E-5 [4.44E6,1.58E-4] | 8.11E-5[3.51E-5,1.28E-4] | | 9.95E-5[4.39E-5,1.58E-4] | ⎯ |
| $\boldsymbol{\nu}_{\boldsymbol{maxfGLNS}}$ | | 6.45E-5[3.86E-5,9.06E-5] | 7.65E-5[5.91E-5,9.36E-5] | | 9.95E-5[8.57E-5,1.18E-4] | ⎯ |
| $\boldsymbol{\nu}_{\boldsymbol{maxrGLNS}}$ | | 3.25E-6[2.49E-6,3.95E-6] | 3.25E-6 [2.71E-6,3.8E-6] | | 1E-5[8.75E-6,1.13E-4] | ⎯ |
| $\boldsymbol{\nu}_{\boldsymbol{maxf GLDH}}$ | | 1.06E-5[5.6E-6, 1.58E-5] | 1.31E-5[6.33E-7,2.56E-5] | | 2.05E-5[7.00E-6,3.14E-5] | ⎯ |
| $\boldsymbol{\nu}_{\boldsymbol{maxr} \boldsymbol{GLDH}}$ | | 2.37E-5 [2.00E-6, 4.5E-5] | 3.61E-5[2.42E-5,4.80E-5] | | 3E-5[2.5E-5,3.80E-5] | ⎯ |
| $\boldsymbol{\nu}_{\boldsymbol{maxf} \boldsymbol{AlaTA}}$ | | 6.00E-5[3.55E-5,8.50E-5] | 6.00E-5 [3.71E-5,8.3E-5] | | 1.15E-4[7.45E-5,1.56E-4] | ⎯ |
| $\boldsymbol{\nu}_{\boldsymbol{maxr AlaTA}}$ | | 1.07E-6 [0,2.53E-6] | 1.22E-6 [0,2.56E-6] | | 1.5E-6[2.01E-7,1.93E-6] | ⎯ |
| $\boldsymbol{\nu}_{\boldsymbol{max GluT}}$ | | 1.28E-6 | 1.65E-6 | | 2.5E-6 | ⎯ |
| $\boldsymbol{\nu}_{\boldsymbol{max resp}}$ | | 7.00E-4[5.61E-4,8.49E-4] | 8.13E-4[5.53E-4,1.00E-3] | | 8.08E-4[6.96E-4,9.20E-4] | ⎯ |
| $\boldsymbol{\nu}_{\boldsymbol{max ATPase}}$ | | 3.60E-3[6.68E-38.53E-3] | 5.86E-3[8.86E-3,1.09E-2] | | 5.80E-3[8.88E-3,1.08E-2] | p<0.1 * ^+^ |
| $\boldsymbol{\nu}_{\boldsymbol{max leak}}$ | | 8.70E-5 | 8.70E-5 | | 8.70E-5 | ⎯ |
| $\boldsymbol{\nu}_{\boldsymbol{maxf CK}}$ | | 2.30E-4 | 2.30E-4 | | 2.30E-4 | ⎯ |
| $\boldsymbol{\nu}_{\boldsymbol{maxr CK}}$ | | 9.00E-5 | 9.00E-5 | | 9.00E-5 | ⎯ |
| $\boldsymbol{\nu}_{\boldsymbol{maxf AK}}$ | | 1.00E-4 | 1.00E-4 | | 1.00E-4 | ⎯ |
| $\boldsymbol{\nu}_{\boldsymbol{maxr AK}}$ | | 3.00E-5 | 3.00E-5 | | 3.00E-5 | ⎯ |
| $\boldsymbol{\nu}_{\boldsymbol{max PPRibP}}$ | | 6.00E-10 | 1.00E-9 | | 1.00E-9 | ⎯ |
| $\boldsymbol{\nu}_{\boldsymbol{max NADPHox}}$ | | 4.00E-3 | 4.00E-3 | | 4.00E-3 | ⎯ |
| $\boldsymbol{\nu}_{\boldsymbol{maxSAL}}$ | 6.65E-6[5.18E-6,8.13E-6] | | 8.54E-6[4.70E-6,1.23E-5] | | 1.00E-5[4.22E-6,1.58E-5] | ⎯ |
| $\boldsymbol{\nu}_{\boldsymbol{maxASX}}$ | 8.05E-6[4.19E-6,1.20E-5] | | 9.91E-6 [3.7E-6,1.63E-5] | | 1.29E-5[5.07E-6,2.08E-5] | ⎯ |
| $\boldsymbol{\nu}_{\boldsymbol{maxfASTA}}$ | 5.41E-6[3.21E-7,1.05E-6] | | 5.65E-6[5.00E-6,6.3E-6] | | 3.50E-5[2.5E-5,4.53E-5] | p<0.1  ^+^ |
| $\boldsymbol{\nu}_{\boldsymbol{maxrASTA}}$ | 3.25E-6 | | 2.16E-6 | | 3.2E-6 | ⎯ |
| $\boldsymbol{\nu}_{\boldsymbol{maxHISARGTA}}$ | 1.50E-5[5.4E-6,2.46E-5] | | 1.85E-5[8.10E-6,2.89E-5] | | 2.05E-5[1.07E-5,3.3E-5] | ⎯ |
| $\boldsymbol{\nu}_{\begin{aligned} \boldsymbol{max} \boldsymbol{LYSILELEU} \\ \boldsymbol{HISVALTYRTA} \end{aligned}}$ | 2.61E-5[1.81E-5,3.43E-5] | | 2.80E-5[1.85E-5,3.75E-5] | | 3.45E-5[2.27E-5,4.65E-5] | ⎯ |
| $\boldsymbol{\nu}_{\boldsymbol{max growth}}$ | 0.48[0.39,0.57] | | 0.52[0.48,0.56] | | 0.43[0.37,0.53] | ⎯ |
| $\boldsymbol{\nu}_{\boldsymbol{max mAb}}$ | 0 | | 1.60E-6[1.05E-6,2.15E-6] | | 1.00E-5[5.55E-6,1.5E-5] p<0.1 * ^+^ | |

Asterisks (*) and (+) denote statistically significant difference between parental and either induced low-producing or induced high-producing cell lines considering shown confidence intervals. All units are in mmol.(10^6^cells)^-1^.h^-1^except for *νmax growth* which is in h^-1^.
